# Hydrophilicity Matching – A Potential Prerequisite for the Formation of Protein-Protein Complexes in the Cell

**DOI:** 10.1371/journal.pone.0011169

**Published:** 2010-06-17

**Authors:** Mario Hlevnjak, Gordan Zitkovic, Bojan Zagrovic

**Affiliations:** 1 Mediterranean Institute for Life Sciences, Split, Croatia; 2 Department of Mathematics, University of Texas at Austin, Austin, Texas, United States of America; 3 Department of Physics, University of Split, Split, Croatia; German Cancer Research Center, Germany

## Abstract

A binding event between two proteins typically consists of a diffusional search of binding partners for one another, followed by a specific recognition of the compatible binding sites resulting in the formation of the complex. However, it is unclear how binding partners find each other in the context of the crowded, constantly fluctuating, and interaction-rich cellular environment. Here we examine the non-specific component of protein-protein interactions, which refers to those physicochemical properties of the binding partners that are independent of the exact details of their binding sites, but which can affect their localization or diffusional search for one another. We show that, for a large set of high-resolution experimental 3D structures of binary, transient protein complexes taken from the DOCKGROUND database, the binding partners display a surprising, statistically significant similarity in terms of their total hydration free energies normalized by a size-dependent variable. We hypothesize that colocalization of binding partners, even within individual cellular compartments such as the cytoplasm, may be influenced by their relative hydrophilicity, potentially in response to local hydrophilic gradients.

## Introduction

From signal transduction cascades to enzymatic activation, from antibody-antigen recognition to cellular trafficking, direct noncovalent protein-protein interactions are the central pillar supporting most of biological functional activity on the molecular level [1]. However, studies of such interactions usually focus on the specifics of the binding sites of the partners, while, at the same time, typically neglect their overall physicochemical properties, with a few notable exceptions at the protein aggregation frontier [2]–[4]. It is generally assumed that binding partners execute random-walk diffusion in a crowded, interaction-rich cellular environment prior to encounter [5]–[7]. Nonetheless, specific interactions that underlie the binding-site recognition itself are all short range and could not serve the purpose of guiding this global, presumably non-specific search for the binding partner.

Moreover, it has been shown that, given low copy numbers and short life-spans of typical signalling proteins in crowded eukaryotic cells, it is imperative that binding partners in signalling cascades be colocalized in order to relay meaningful signals on reasonable time scales [8], [9]. It is known that proteins colocalize due to segregation into different organelles or cellular compartments, sequestration via anchor and scaffold proteins, or sometimes even chemical modifications [1]. For example, interactions between two membrane proteins are greatly facilitated by both of them being colocalized in the 2D-membrane, which is easier to search by diffusion [9], [10]. In this case, almost trivially, the finding of the binding partners is enabled by a non-specific element encoded in their respective structures – the hydrophobicity of their overall molecular surface. Importantly, this non-specific component of protein-protein interactions may not be related to the specific features of the complementary binding sites of two proteins, and still significantly influence the binding. However, little attention has been paid to such general mechanisms when it comes to cytoplasmic or nucleoplasmic proteins, with some notable exceptions [11]–[13]. For example, significant commonalities were found for isoelectric points of proteins assigned to different nuclear compartments [11], [12], or for the pH of maximal stability of a complex and its monomers [14]. Nevertheless, the majority of these studies focused on the information encoded in the sequence of colocalized proteins, and not necessarily in their 3D-structure. Here we examine whether any signature of potential colocalization mechanisms for a large set of known binding partners is encoded in their 3D-structures by searching for commonalities between partners in the same complex.

As a source of 3D-structures of known cocrystallized interacting partners, we used the DOCKGROUND database of transient, binary protein complexes in their unbound form [15]. After performing additional short relaxation molecular dynamics (MD) simulations of each of the binding partners, we evaluated for each of them different geometric properties such as solvent-accessible surface area, radius of gyration, and volume, or different physicochemical properties such as total charge, isoelectric point, hydration free energy (HFE), and total electrostatic energy (EE). We quantified the degree of similarity of the binding partners by calculating intraclass correlation coefficients (ICCs) [16], [17] for different properties, and evaluated the associated p-values via randomization tests.

## Results and Discussion

Pairs of interacting partners were classified into different subsets based on their origin and the known site of complex formation in the cell or extracellular space, following the detailed characterization of the entire set of 268 proteins (Table S1). We focus first on the subset containing 118 eukaryotic proteins (59 pairs) interacting in the cytoplasm or nucleoplasm. Similar results were obtained for a larger subset comprised of 162 proteins (81 pair), including additionally also archeal and bacterial proteins, or for the complete set containing 268 proteins (134 pairs), including intra- or extracellular segments of transmembrane proteins, as well as organellar and secreted proteins (Table 1, Text S1, Fig. S3, Fig. S4 and Fig. S5).

**Table 1:** Summarized results showing the degree of similarity of the known binding partners for various properties within different subsets.

	59 pairs*		81 pair†		53 pairs‡		134 pairs§	
compared property	ICC	p-value	ICC	p-value	ICC	p-value	ICC	p-value
N	0,462	0,682075	0,408	0,953692	0,418	0,877154	0,439	0,919747
R_gyr_	0,616	0,035292	0,525	0,293705	0,409	0,900328	0,482	0,634996
SASA	0,484	0,553248	0,409	0,953584	0,421	0,870251	0,424	0,965246
vol	0,462	0,683424	0,411	0,947762	0,421	0,867210	0,436	0,930065
HFE	0,488	0,526324	0,454	0,777910	0,443	0,772098	0,450	0,869443
EE	0,489	0,524100	0,437	0,864386	0,440	0,788886	0,464	0,781681
HFE/N	**0,777**	**0,000055**	**0,737**	**0,000155**	0,545	0,229343	**0,761**	**0,000001**
HFE/R_gyr_	0,517	0,355147	0,505	0,429767	0,490	0,527236	0,515	0,344223
HFE/SASA	**0,710**	**0,000593**	0,697	0,001329	0,544	0,241198	**0,708**	**0,000013**
HFE/vol	**0,773**	**0,000109**	**0,720**	**0,000735**	0,495	0,494577	**0,739**	**0,000009**
EE/N	**0,798**	**0,000001**	**0,762**	**0,000065**	0,552	0,201419	**0,720**	**0,000015**
EE/R_gyr_	0,532	0,287528	0,500	0,477764	0,476	0,603419	0,512	0,374598
EE/SASA	0,643	0,010989	0,656	0,001747	0,475	0,611896	0,623	0,001726
EE/vol	**0,805**	**0,000002**	**0,771**	**0,000024**	0,584	0,096769	**0,747**	**0,000001**

* eukaryotic intracellular (nuclear and cytosolic) complexes.

† archeal, bacterial and eukaryotic intracellular (nuclear and cytosolic) complexes (includes the entire subset of 59 binary complexes).

‡ archeal, bacterial and eukaryotic extracellular complexes, or intracellular complexes of organellar proteins or segments of transmembrane proteins.

§ maximal set comprised of ^†^ and ^‡^ ; the p-values<0.001 are shown in bold.

**Figure 1:**
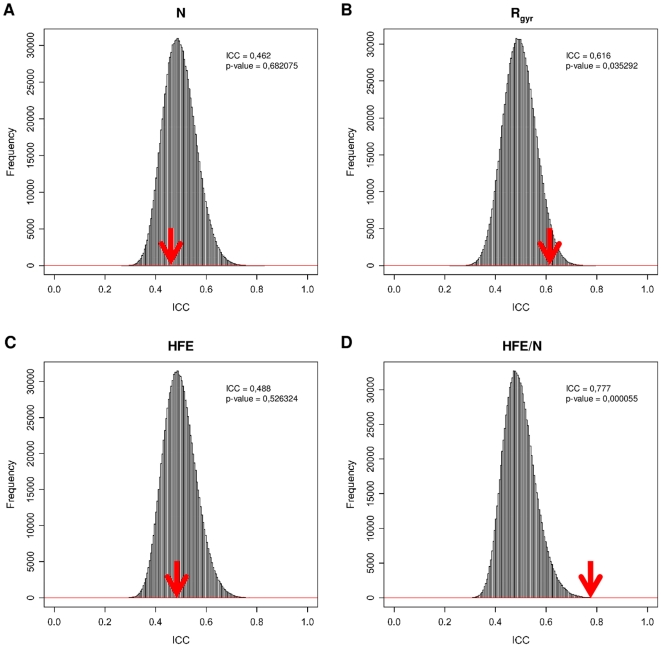
Comparison of ICCs calculated for naturally occuring binding partners and those obtained by a randomization procedure. The results are for a subset of 118 eukaryotic proteins (59 pairs) that interact in the cytoplasm or nucleoplasm. The ICC values were calculated for (A) the sequence length of the binding partners (N), (B) their radius of gyration (R_gyr_), (C) hydration free energy (HFE), and (D) HFE normalized by sequence length (HFE/N). Red arrow denotes the value of the observed ICC for the known binding partners.

If the known binding partners are compared with respect to the sequence length (N) of the fragments found in cocrystallized complexes (Fig. 1A), they expectedly exhibit no similarity whatsoever. The observed ICC of 0.462 and the associated p-value of 0.682075 mean that the same degree of similarity occurs in 68% of the cases where the pairs are chosen completely at random from the studied subset. It is important to note that the majority of cocrystallized proteins, including those that were examined herein, are fragments of larger proteins. For example, within the subset of 118 eukaryotic proteins, the average completeness of their structures is around 50% (Table S1). Even so, one observes a significantly higher similarity between the binding partners with respect to their radii of gyration, which occurs by chance in only 3.5% of the cases (Fig. 1B). It is possible that the observed matching is a consequence of the experimental procedure that complexes were subjected to: it can be that a match in radius of gyration could help packing of the partners in the crystal during cocrystallization. However, we do not observe such matching in the remaining analyzed subsets, which speaks against this speculation (Table 1). Comparison of the binding partners with respect to their HFEs, as calculated by GB/SA methodology [18], [19], does not reveal any significant similarity between them (Fig. 1C). Surprisingly, when their HFEs are normalized by either their respective sequence length (Fig. 1D), or volume (Fig. S3A), the binding partners show highly significant similarity, which itself occurs by chance in a remarkable one out of eighteen thousand cases (p-value of 0.000055). This finding is further illustrated by a symmetric scatter plot of the data in question (Fig. S8). Finally, size-normalized electrostatic energy also appears to be significantly matched between partners (p-value of 10^−6^), while other calculated geometric properties, such as volume or solvent-accessible surface area exhibit significantly lower levels of matching in this subset (Table 1).

**Figure 2:**
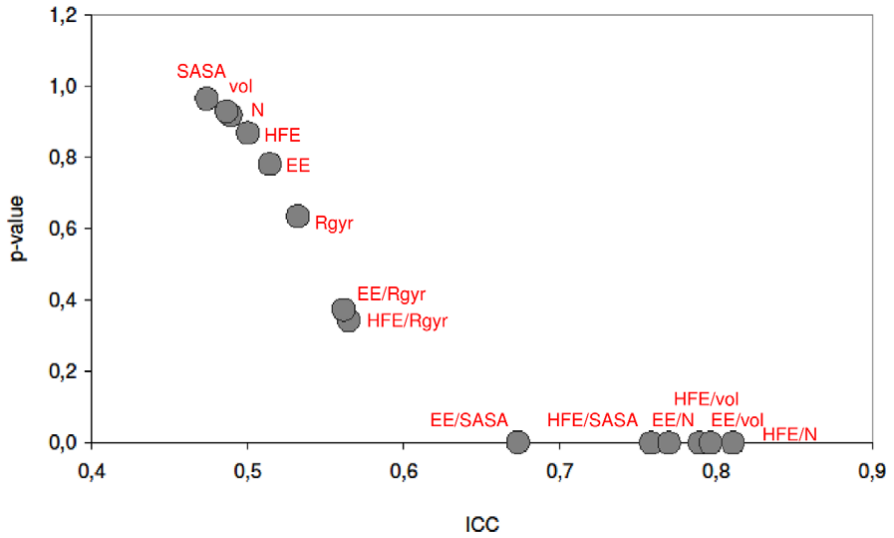
Summary of the calculated ICCs and their associated p-values for various properties. The results are for the maximal set of 268 proteins (134 pairs). We show the results for amino acid sequence length (N), volume (vol), radius of gyration (R_gyr_), solvent-accessible surface area (SASA), hydration free energy (HFE), and electrostatic energy (EE), or selected ratios thereof.

**Figure 3:**
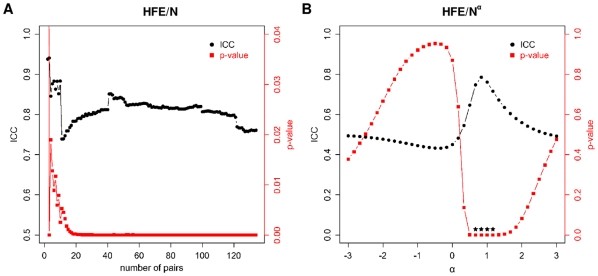
ICCs and p-values for the HFE/N^α^ ratio for the maximal set of 268 proteins (134 pairs). This set includes organellar, secreted, and extra- or intracellular fragments of transmembrane proteins. The values are plotted as a function of (A) the subset size, where the pairs were ordered by their maximal length (maximal N within a given pair) for α = 1, and (B) the exponent α, with the scan performed in steps of 1/6. Stars denote the p-values<0.0001.

Interestingly, when expanding this set by including organellar and extracellular proteins, or even cytoplasmic fragments of transmembrane proteins, a similar trend of matching properties is observed (Fig. 2). For example, the HFE normalized by sequence length, solvent accessible surface area or volume, remains well matched regardless of the set increasing in size from 59 to 81 or 134 pairs of proteins (Table 1, Fig. 3A). For the complete data set (134 pairs), in fact, the statistical significance of intra-pair matching for HFE/N reaches a maximum with an ICC of 0.761 and a p-value of 10^−6^ (Table 1, Fig. 2). A similar situation is observed for size-normalized electrostatic energy (Fig. 2, Table 1), which is not surprising, as HFE and EE are closely related. Namely, in the GB/SA formalism, the polar, electrostatic part is the major component of HFE, and the correlation coefficient between the two for all of the proteins in our data set is R = 0.77 (Fig. S9). Finally, when proteins that are known to be specifically directed to a certain intra- or extracellular location (via some sort of signal sequence) are analyzed separately, no match in their size-normalized HFEs or any other property we examined is observed (Table 1, 53 pair set, Fig. S5B, Text S1).

Even though hydration free energies, when normalized by radii of gyration, do not seem to be significantly matched between the partners regardless of the data set used (Table 1, Fig. S3B), we noticed that for small proteins (where both of the partners have less than 130 residues), this ratio seems to be extremely well correlated (ICCs above 0.9) between the members of the pair (Fig. S6). This type of size-filtering resulted in either 24 complexes from the set of all intracellular proteins (subset of 81 pairs), or 28 complexes when extracted from the maximal set (134 pairs). When including also proteins up to 150 residues in our analysis, their similarity in the abovementioned property decreases, but nonetheless stays very high, with ICCs around 0.8 (data not shown).

Overall, the necessity for normalizing the HFEs by a size-dependent term is further emphasized if one examines the behavior of HFE/N^α^ for a range of exponents α (Fig. 3A). Clearly, the most significant match between the partners is observed only for a narrow range of such exponents, surrounding 1 (cca. 0.5–1.2). The most obvious rationale for normalization of HFE by a size-dependent term is to adjust for missing residues, since HFEs depend on the size of proteins. In this way, size as a variable is eliminated, and the partners that are being compared are set on an equal footing. Another possibility is illustrated by considering a mixture of small and large proteins that have the same HFEs and are competing for the same compartment characterized by a given level of hydrophilicity. Here, the smaller proteins would likely prevail since more of them could fit in this compartment, and as a consequence, size-normalized HFE would be the pertinent variable to be matched. However, if one looks at complexes in our data set where both partners are complete (17 complexes in total), one sees no significant matching for size-normalized HFE, weakening the latter argument (Fig. S7, Text S1). Future research should elucidate a rigorous physical basis for matching of size-normalized HFE. Interestingly, a similar strategy is used in prediction of protein retention times in hydrophobic interaction chromatography, where hydrophobicity is normalized by solvent-accessible surface area [20], [21], also a size-dependent variable.

Analysis of isoelectric points and charges at neutral pH estimated from primary sequences did not reveal any statistically significant trends (Fig. S2A and Fig. S2B), except to a moderate degree when charge is normalized by N (p-values of 0.01, Fig. S2C and Fig. S2D, Text S1). However, splitting the solvent-accessible surface area (SASA) into positively and negatively charged regions, as well as into hydrophilic and hydrophobic regions (refer to Materials and Methods for details), and comparing these regions between the partners further supports the above findings (Table S3 and S4). Firstly, when absolute values of different types of SASA are compared between partners, no significant matching is observed (Table S3). On the other hand, when different types of SASA are compared after normalization by the total SASA, significant level of matching is observed for positively charged and total charged SASA, as well as for hydrophilic and hydrophobic SASA for different subsets (Table S4). Apparently, regardless of how one quantitates size-normalized hydrophilicity, the matching between known partners reaches statistically very significant levels. Furthermore, size-normalized total charge, the same as the size-normalized electrostatic energy discussed above, is closely related to protein's hydrophilicity, and it is not surprising that analogous levels of matching are seen here as well.

**Figure 4:**
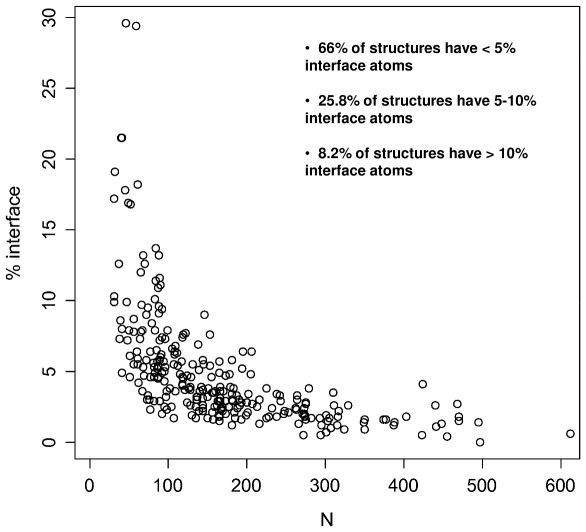
Fraction of atoms comprising the interface between each of the partners. Size of the interface as a function of the sequence length of partners (N) is shown for the maximal set of 268 proteins (134 pairs).

It is possible that the observed matched properties, such as the size-normalized HFE, are significantly influenced by the properties of the binding sites themselves, which in turn, almost by definition have certain properties in common, such as the solvent-accessible surface area. To exclude this possibility, we calculated the fraction of atoms that form the binding-site interface for each protein. Given the fact that the size of the interface for the majority of proteins used in this analysis is below 10% of the total number of atoms (Fig. 4), we assume that the contribution of the interface itself to the calculated properties is not responsible for the correlations observed. Alternatively, it is possible that the observed matching may be a consequence of the experimental treatment that proteins underwent prior to crystallization or the consequence of the cocrystallization experiment itself. In other words, our dataset might be biased with respect to those complexes that are more readily cocrystallized, which in turn might be precisely those complexes whose constituents are matched in size-normalized HFE or some other property discussed above. An obvious example of such bias are, for example, intrinsically unfolded proteins [22], which are, by definition, absent from structural databases. Currently, it is not possible to fully discount this possibility, but if true, it might be exciting in its own right, especially in the context of assessing crystalizability of different complexes and designing structural experiments.

With a recent report showing that the localization of a bacterial protein is determined by a geometric factor [23], [24], namely, membrane curvature, the importance of assessing potential contribution of other non-specific properties to protein localization in the cell is additionally emphasized. Proteins are physicochemical entities, and the fact that their localization and interactions are exclusively determined by the particulars of the specific binding sites (to either their partners or anchoring elements such as cytoskeleton), as typically assumed [25], needs to be rigorously tested. The results presented here indicate that binding partners in different transient functional complexes have certain general physicochemical properties in common, which could then be responsible for their colocalization or clustering on the microscopic level, and thus indirectly facilitate their binding. Our results suggest that size-normalized HFE may be one such property, and allow us to propose the hydrophilicity matching hypothesis, where putative hydrophilic gradients, almost as in chromatographic separation [20], [21], may serve as an organizing force for the localization of proteins, even within individual compartments such as the cytoplasm. Whether proteins themselves can generate such gradients remains to be explored. A similar proposal about the origins of microcompartmentation in the cytoplasm was made some time ago by Walter and Brooks [26]. 

**Figure 5:**
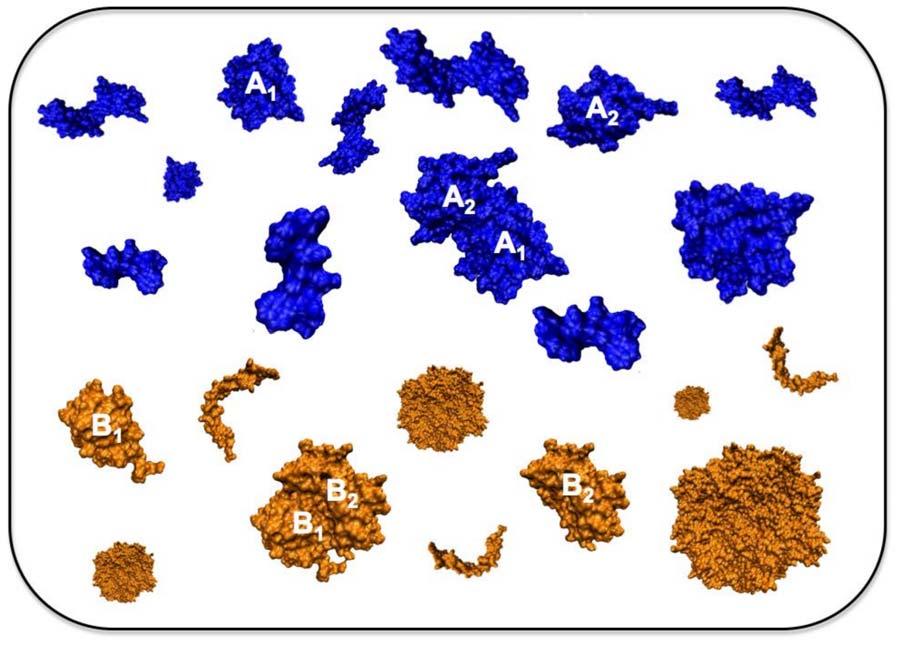
Schematic representation of the hydrophilicity matching hypothesis. Blue and orange encodes for proteins that are hydrophilic to different extent, and hence colocalize to different regions of the cell. Proteins that are meant to interact (A1 and A2, and B1 and B2) exhibit matching levels of hydrophilicity (HFE/N) and are therefore colocalized.

It is our belief that *protein ecology* – where a given protein is located, and who and for what reasons its molecular neighbours are, even within individual compartments – may be an important frontier to study (Fig. 5). Should it really turn out that the non-specific component of protein-protein interactions is functionally relevant, and therefore also under evolutionary control, this would represent a major paradigm shift, and would carry important implications on how we view biological systems on the molecular level or try to affect them in practical situations. For example, most drug design applications almost exclusively target the specific component of protein-protein interactions. Should the non-specific component prove to be relevant, it would also present itself as a completely novel, orthogonal pharmacological target.

## Materials and Methods

### Dataset

The DOCKGROUND database [15] used herein contains either experimentally determined structures of the binding partners in their unbound form (when available), or the ones that are computationally modelled based on bound complexes. The starting set of 151 binary complexes obtained by excluding all members of the database (release of 8^th^ July 2008) with missing atoms anywhere in the backbone, was reduced to 134 after all non-physiological complexes (antibody-antigen complexes that do not exist *in vivo* or artificially created proteins) were excluded. The completeness of each of the partners was determined by taking the ratio of the number of residues of a given protein in the cocrystallized complex and the length of the native protein as reported in the UniProt database. Signal peptides, as defined within the UniProt database, were excluded when calculating the completeness in those cases where they were present. Structures were considered to be complete if 3% of the residues or less were missing in the cocrystallized complex. Localization of proteins was determined by an exhaustive literature research combined with the information available in the UniProt database. Localization of proteins based on where the encounter with their respective partners takes place was found to differ in some cases from their general localization as reported in UniProt or other databases. Viral proteins were assigned origin and grouped based on the characteristics of their interacting partner.

### Calculation of physicochemical properties

Structures were prepared for calculation using PDB2PQR software (version 1.3) [27]. The volume of interacting partners (vol) was calculated using 3v: Voss Volume Voxelator (version 1.2) [28], solvent-accessible surface area (SASA) using DSSP [29], while hydration free energy (HFE), electrostatic energy (EE) and radius of gyration (R_gyr_) were calculated using TINKER molecular modeling package (version 4.2) [30]. Average values of properties were obtained from an ensemble of 100 structures generated via additional short relaxation MD simulations of each member of the pair (10 ps of total simulated time per protein). Simulations were run in implicit GB/SA solvent with Langevin dynamics at 300 K, using OPLSaa force field [31] with no cutoffs for electrostatics, and friction coefficient of γ = 91 ps^−1^. HFE was calculated using GB/SA methodology [18], [19] with ε_water_ = 81. Isoelectric point values and charges at neutral pH were estimated using web-based Protein Calculator v3.3 (http://www.scripps.edu/~cdputnam/protcalc.html). Different types of SASA (positively charged, negatively charged, total charged, hydrophilic and hydrophobic) were calculated with GROMACS (version 4.0.5) [32] using the g_sas subroutine. Default settings of the g_sas subroutine were used for discriminating hydrophilic and hydrophobic SASA, while positively charged SASA was defined as exposed lysine and arginine, and negatively charged SASA as exposed aspartate and glutamate residues. The fraction of the atoms that form the interface between the partners when in their bound state was determined by counting atoms of each of the partners whose distance was smaller than the sum of their respective van der Waals radii plus an arbitrary value of 0.5 Å. Van der Waals radii used are as follows: r_vdW_(C) = 1.7 Å, r_vdW_(N) = 1.55 Å, r_vdW_(O) = 1.52 Å, r_vdW_(S) = 1.8 Å [33], r_vdW_(H) = 1.09 Å [34].

### Statistics

Intraclass correlation coefficients (ICCs) for a particular property were determined as previously reported [16], [17]. Intraclass correlation is a standard statistical test for quantifying the extent to which the members of a given group resemble each other in terms of a certain property. For paired data sets where there is no meaningful way of ordering members of a given pair (such as properties of twins, for instance), ICC represents a more natural measure of association than the Pearson correlation coefficient (R), which is typically reserved for those cases where there is a clear distinction between dependent and independent variables. In order to illustrate this difference, average Pearson correlation coefficient estimates for various properties between binding partners in different analyzed subsets are additionally discussed in the Supporting Information (Text S1, Fig. S1 and Table S2).

For a paired data set comprised of N pairs,
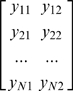
the group mean 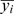, the total mean 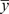, the variance between the groups 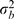 and the variance within the groups 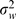 are given as
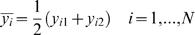
(1)

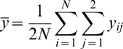
(2)

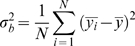
(3)

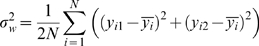
(4)respectively. Then, the corresponding ICC (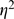) is defined as:
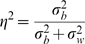
(5)ICC captures the relation between the average variance within pairs and the total variance between pairs. The significance of the observed ICCs was assessed via randomization tests in which proteins within a given set were paired completely at random, to obtain a same-size, randomized set of pairs whose ICC value was then calculated. After 10^6^ such randomizations, the associated p-values were estimated by calculating the fraction of times an ICC value that is greater than or equal to the one for the native data set appeared in the distribution of ICCs for randomized sets.

## Supporting Information

Text S1 A thorough discussion of various analyzed properties within protein subsets obtained by filtering of the maximal set using different criteria.
(0.04 MB DOC)

Table S1 Characteristics of the maximal set (134 complexes in total).
(0.35 MB DOC)

Table S2 Average Pearson correlation coefficient estimates <R> for various properties between binding partners in different subsets.
(0.04 MB DOC)

Table S3 Comparison of the absolute values (nm^2^) of different types of solvent-accessible surface area (SASA) between the known binding partners within different subsets.
(0.03 MB DOC)

Table S4 Comparison of different types of solvent-accessible surface area (SASA) normalized by total SASA between the known binding partners within different subsets.
(0.03 MB DOC)

Figure S1 ICC vs Pearson R for various calculated properties. We show only the data points with ICC>0.5, indicating positive correlation in terms of R. The plotted R is the average obtained by 10^6^ permutations of the members of each pair.
(0.10 MB TIF)

Figure S2 Comparison of ICCs calculated for naturally occuring binding partners and those obtained by a randomization procedure. The results are for the entire set of 268 eukaryotic proteins (134 pairs). The ICC values were calculated for (A) isoelectric point values (pI), (B) absolute values of charge (|charge|), (C) charge normalized by sequence length (charge/N), and (D) absolute values of charge normalized by sequence length of the partners (|charge|/N). The values of charge used were all at neutral pH. Red arrow denotes the value of the observed ICC for the known binding partners.
(0.22 MB TIF)

Figure S3 Comparison of ICCs calculated for naturally occuring binding partners and those obtained by a randomization procedure. The results are for a subset of 118 eukaryotic proteins (59 pairs) that interact in the cytoplasm or nucleoplasm. The ICC values were calculated for (A) the hydration free energy normalized by volume of the partners (HFE/vol), (B) HFE normalized by radius of gyration (HFE/R_gyr_), (C) electrostatic energy normalized by sequence length (EE/N), and (D) electrostatic energy normalized by volume (EE/vol). Red arrow denotes the value of the observed ICC for the known binding partners.
(0.29 MB TIF)

Figure S4 Comparison of ICCs calculated for naturally occuring binding partners and those obtained by a randomization procedure. The results are for a subset of 162 intracellular proteins from all three domains of life. The ICC values were calculated for (A) the radius of gyration (R_gyr_), (B) hydration free energy (HFE), (C) HFE normalized by sequence length (HFE/N), and (D) HFE normalized by volume (HFE/vol). Red arrow denotes the value of the observed ICC for the known binding partners.
(0.24 MB TIF)

Figure S5 Comparison of ICCs calculated for naturally occuring binding partners and those obtained by a randomization procedure. The ICC values were calculated for the size-normalized hydration free energy (HFE/N) in (A) the maximal set with all analyzed proteins (268 proteins in total), and (B) set containing only organellar and secreted proteins, as well as intra- and extracellular segments of transmembrane proteins (106 proteins in total). Red arrow denotes the value of the observed ICC for the known binding partners.
(0.14 MB TIF)

Figure S6 Comparison of ICCs calculated for naturally occuring binding partners and those obtained by a randomization procedure. The ICC values were calculated for the hydration free energy normalized by radius of gyration of the partners (HFE/R_gyr_) for (A) 24 complexes, and (B) 28 complexes. Complexes were extracted by size-filtering of fragmented proteins with a criterion that both of the partners have less than 130 residues. Red arrow denotes the value of the observed ICC for the known binding partners.
(0.21 MB TIF)

Figure S7 Comparison of ICCs calculated for naturally occuring binding partners and obtained by a randomization procedure. The results are for the set of complete proteins (17 complexes). The ICC values were calculated for (A) the hydration free energy normalized by sequence length (HFE/N), and (B) hydration free energy normalized by volume of the partners (HFE/vol). Red arrow denotes the value of the observed ICC for the known binding partners.
(0.24 MB TIF)

Figure S8 Symmetric scatter plot of the size-normalized hydration free energy (HFE/N). The data shown is for a subset of 118 eukaryotic proteins (59 pairs) that interact in the cytoplasm or nucleoplasm. Because it is impossible to uniquely assign each member of a given pair to either x or y axes, here we show both (x,y) and (y,x) possibilities for each point.
(0.15 MB TIF)

Figure S9 Electrostatic energy (EE) vs hydration free energy (HFE). The values shown are average HFE and EE calculated for all analyzed proteins (268 proteins in total).
(0.18 MB TIF)
