# Supplementary material for: Hydrophilicity Matching – A Potential Prerequisite for the Formation of Protein-Protein Complexes in the Cell
Source: PLoS One. 2010 Jun 17;5(6):e11169. doi: 10.1371/journal.pone.0011169 (PMC2887369; doi:10.1371/journal.pone.0011169)
Supplement: Text S1 — A thorough discussion of various analyzed properties within protein subsets obtained by filtering of the maximal set using different criteria. (0.04 MB DOC) [file pone.0011169.s001.doc]

**Supporting Information Text S1**

**Hydrophilicity Matching – a Potential Prerequisite for the Formation of Protein-Protein Complexes in the Cell**

**Mario Hlevnjak^1,2^, Gordan Zitkovic^3^ & Bojan Zagrovic^1,2,4*^**

^1^Mediterranean Institute for Life Sciences

Split, Croatia

^2^Laboratory of Computational Biophysics

Department of Structural and Computational Biology

Max F. Perutz Laboratories GmbH

Vienna, Austria

(present address for MH and BZ)

^3^Department of Mathematics

University of Texas at Austin

Austin, Texas, United States of America

^4^Department of Physics

University of Split

Split, Croatia

*To whom correspondence should be addressed: Bojan Zagrovic, Laboratory of Computational Biophysics, Department of Structural and Computational Biology, Max F. Perutz Laboratories GmbH, Campus Vienna Biocenter 5, A-1030 Vienna, Austria, Phone: +43-1-4277-52271, Fax: +43-1-4277-9522, E-mail: bojan.zagrovic@univie.ac.at

**Statistics**

*ICC vs. Pearson R*

In addition to determining ICCs in our analysis, we also estimated the Pearson correlation coefficient for different properties by randomly assigning the members of each pair to either an independent or a dependent variable, and subsequently averaging the correlation coefficients that we obtained in this way over 10^6^ such randomized permutations (Table S2). When plotting ICCs that were calculated for various properties in all of the subsets in our analysis versus their estimated average Pearson Rs, one observes a remarkably linear relationship between the two (Fig. S1). The obtained linear relationship suggests that, although intuitively a better measure of the strength of association between the partners, ICC is likely to be sensitive to outliers within the set to a similar extent as R – the difference being only in the way in which the association is estimated. Here we illustrate that such estimates of R, regardless of being modest in size, still display a striking statistical significance in terms of the p-values that are associated with their corresponding ICCs (Table S2 vs Table 1).

**Results for different analyzed subsets**

*Isoelectric point and charge analysis*

The ICC analysis does not reveal any significant match between the binding partners in terms of their isolectric point values or the absolute value of their charge at pH = 7 (Fig. S2A, and Fig. S2B). On the other hand, when charge is normalized by the sequence length of the partners, a peculiar trend is observed. Namely, binding partners seem to be weakly anticorrelated with respect to their size-normalized total charge (Fig. S2C), while at the same time significantly correlated or matched, when it comes to their size-normalized absolute charge values (Fig. S2D).

We can only speculate that a stronger signal, showing that the partners are matched in their size-normalized charge, might be observed using data of higher quality, instead of data obtained based on protein sequences alone.

*ICC analysis for all eukaryotic intracellular proteins (59 pairs)*

The subset of 118 proteins (59 complexes) was generated by including only those eukaryotic intracellular proteins that interact in the cytoplasm or in the nucleus, since our main hypothesis that colocalization of interacting partners might be influenced by their hydrophilicity, is primarily concerned with this set. This set has not been further divided due to the difficulties in distinguishing exclusively nuclear from exclusively cytoplasmic proteins, especially given the fact that many shuttle back and forth between these two compartments, and also because many of the proteins can enter nucleus without being specifically directed there. The most important results pertaining to this set were presented in the main article. Here we show the individual randomization distribution of ICCs (Fig. S3) for several selected properties that were mentioned in the main article. The remainder of the results for this and all other subsets is summarized in Table 1.

*ICC analysis for all eukaryotic, bacterial and archeal intracellular proteins (81 pairs)*

After the previous set was expanded by including also those archeal and bacterial intracellular proteins, which, presumably, are not specifically directed to their site of interaction via some sort of a signal sequence (to an organelle, a membrane or extracellular space), the resulting set increased to 162 proteins (81 complex) (Table S1). The results obtained are quite similar to those already observed in the smaller subset of 118 proteins (59 complexes) (Table 1), so here we show the individual randomization distribution of ICCs (Fig. S4) for the same properties that were discussed in the main article for the smaller subset.

*ICC analysis for the maximal set (134 pairs)*

As mentioned in the main article, an additional set expansion from 162 to 268 proteins by inclusion of the intracellular segments of transmembrane proteins, as well as extracellular or secreted ones, resulted in an undeniably strong degree of matching of the size-normalized hydration free energy between the partners (Fig. S5A). When analyzed separately, this newly included set of 106 proteins, all of which are specifically directed to a certain intra- or extracellular location, fails to show a significant match in HFE/N between the partners (Fig. S5B).

When filtering of the maximal set is performed based on the completeness of available structures, there are only 17 complexes with both partners complete, 7 of which are intracellular only. Important to note, we fail to detect any significant matching of the partners with respect to their hydration free energies, regardless of the way it is normalized (Fig. S7). Given the hypothesis that colocalization of interacting partners might be influenced by their hydrophilicity, apparently, it is hard to reconcile these observations for the complete partners (Fig. S7) with the previously obtained ones in which the hydration free energy was normalized either by sequence length of the partners or volume, where the similarity of partners in terms of these properties is highly statistically significant (Fig. S4C, Fig. S4D, and Table 1).

One of the underlying reasons for apparent discrepancy of the results obtained for complete versus incomplete proteins might be the size of the dataset of complete proteins (17 complexes), which is, in this case, 3 to 8 times smaller when compared to previously analyzed subsets. An important argument in defense of our hypothesis would be the composition of this reduced set. More than a half of it is comprised of proteins which are specifically targeted to their destination via some sort of signal sequence (to an organelle, a membrane or extracellular space), and since the subset of such proteins fails to show any significant match in HFE/N, as previously mentioned (Fig. S5B), it is no surprise that the subset of complete proteins behaves similarly. Regardless of the underlying reasons, this observed discrepancy should serve as an additional motivation for a proper experimental verification of the proposed *hydrophilicity matching* hypothesis.
