# Supplementary material for: Hydrophilicity Matching – A Potential Prerequisite for the Formation of Protein-Protein Complexes in the Cell
Source: PLoS One. 2010 Jun 17;5(6):e11169. doi: 10.1371/journal.pone.0011169 (PMC2887369; doi:10.1371/journal.pone.0011169)
Supplement: Table S1 — Characteristics of the maximal set (134 complexes in total). (0.35 MB DOC) [file pone.0011169.s002.doc]

Table S1. Characteristics of the maximal set (134 complexes in total).

| **PDB ID** | **N residues 1^st^** | **N residues 2^nd^** | **% completeness 1^st^** | **% completeness 2^nd^** | **% interface 1^st^** | **% interface 2^nd^** | **localization 1^st^** | **localization 2^nd^** | **origin 1^st^** | **origin 2^nd^** |
| --- | --- | --- | --- | --- | --- | --- | --- | --- | --- | --- |
|  |  |  |  |  |  |  |  |  |  |  |
| **1a2x** | 158 | 31 | complete | 17 | 3,5 | 17,2 | cytoplasm | cytoplasm | eukaryote (rabbit) | eukaryote (rabbit) |
| **1a9n** | 162 | 94 | 64 | 42 | 2,6 | 5,7 | nucleus | nucleus | eukaryote (human) | eukaryote (human) |
| **1agr** | 350 | 128 | complete | 62 | 1,6 | 3,7 | cytoplasm | cytoplasm | eukaryote (rat) | eukaryote (rat) |
| **1ais** | 193 | 181 | 64 | 95 | 1,6 | 1,2 | cytoplasm | cytoplasm | archea (pyrococcus) | archea (pyrococcus) |
| **1ava** | 403 | 181 | complete | complete | 1,8 | 3,4 | secreted, extracellular | secreted, extracellular | eukaryote (barley) | eukaryote (barley) |
| **1ay7** | 96 | 89 | complete | complete | 5,3 | 5,8 | secreted, extracellular | cytoplasm | bacteria (streptomyces) | bacteria (bacillus) |
| **1bh9** | 89 | 45 | 42 | 36 | 11,6 | 17,8 | nucleus | nucleus | eukaryote (human) | eukaryote (human) |
| **1blx** | 305 | 160 | 94 | 96 | 1,7 | 3,6 | cytoplasm | cytoplasm | eukaryote (human) | eukaryote (mouse) |
| **1bui** | 247 | 121 | 31 | 89 | 2,0 | 3,7 | secreted, extracellular | secreted, extracellular | eukaryote (human) | virus (bacteriophage) |
| **1bvn** | 495 | 74 | complete | complete | 1,4 | 9,5 | secreted, extracellular | secreted, extracellular | eukaryote (pig) | bacteria (streptomyces) |
| **1c1y** | 167 | 77 | 92 | 12 | 2,3 | 6,4 | cytoplasm | cytoplasm | eukaryote (human) | eukaryote (human) |
| **1c4z** | 350 | 144 | 40 | 94 | 0,9 | 3,1 | cytoplasm | cytoplasm | eukaryote (human) | eukaryote (human) |
| **1cf7** | 82 | 67 | 18 | 16 | 2,9 | 3,6 | nucleus | nucleus | eukaryote (human) | eukaryote (human) |
| **1ci6** | 56 | 47 | 16 | 16 | 7,8 | 9,9 | nucleus | nucleus | eukaryote (human) | eukaryote (mouse) |
| **1clv** | 470 | 32 | complete | complete | 1,5 | 19,1 | cytoplasm | cytoplasm | eukaryote (yellow mealworm) | eukaryote (amaranthus) |
| **1cxz** | 182 | 86 | 96 | 9 | 2,9 | 5,7 | cytoplasm | cytoplasm | eukaryote (human) | eukaryote (human) |
| **1d2z** | 150 | 102 | 32 | 20 | 3,2 | 3,8 | cytoplasm | cytoplasm | eukaryote (fruit fly) | eukaryote (fruit fly) |
| **1d3b** | 72 | 81 | 57 | 34 | 5,8 | 5,6 | nucleus | nucleus | eukaryote (human) | eukaryote (human) |
| **1dev** | 194 | 41 | 42 | 3 | 5,2 | 21,5 | cytoplasm | cytoplasm | eukaryote (human) | eukaryote (human) |
| **1ds6** | 181 | 179 | 96 | 89 | 3,0 | 3,9 | cytoplasm | cytoplasm | eukaryote (human) | eukaryote (human) |
| **1dtd** | 303 | 61 | complete | 92 | 1,5 | 5,9 | secreted, extracellular | secreted, extracellular | eukaryote (human) | eukaryote (leech) |
| **1e44** | 96 | 84 | 17 | complete | 7,3 | 11,4 | cytoplasm | cytoplasm | bacteria (escherichia) | bacteria (escherichia) |
| **1ebd** | 455 | 41 | complete | 10 | 0,4 | 4,9 | cytoplasm | cytoplasm | bacteria (bacillus) | bacteria (bacillus) |
| **1eer** | 213 | 166 | 44 | complete | 2,5 | 3,9 | extracellular | secreted, extracellular | eukaryote (human) | eukaryote (human) |
| **1ewy** | 295 | 98 | 67 | complete | 1,2 | 3,6 | cytoplasm | cytoplasm | bacteria (anabaena) | bacteria (anabaena) |
| **1f02** | 282 | 66 | 30 | 12 | 1,7 | 9,7 | extracellular | secreted, extracellular | bacteria (escherichia) | bacteria (escherichia) |
| **1f60** | 440 | 90 | 96 | 44 | 2,6 | 11,1 | cytoplasm | cytoplasm | eukaryote (yeast) | eukaryote (yeast) |
| **1f6m** | 316 | 108 | complete | complete | 1,8 | 6,2 | cytoplasm | cytoplasm | bacteria (escherichia) | bacteria (escherichia) |
| **1fbv** | 388 | 144 | 43 | 94 | 1,4 | 3,7 | cytoplasm | cytoplasm | eukaryote (human) | eukaryote (human) |
| **1ffg** | 128 | 68 | complete | 10 | 2,6 | 5,3 | cytoplasm | cytoplasm | bacteria (escherichia) | bacteria (escherichia) |
| **1fm9** | 232 | 272 | 50 | 54 | 2,4 | 0,5 | nucleus | nucleus | eukaryote (human) | eukaryote (human) |
| **1fr2** | 131 | 83 | 23 | complete | 5,5 | 7,9 | cytoplasm | cytoplasm | bacteria (escherichia) | bacteria (escherichia) |
| **1g3n** | 293 | 155 | 90 | 92 | 1,7 | 4,6 | cytoplasm | cytoplasm | eukaryote (human) | eukaryote (human) |
| **1g73** | 157 | 89 | 85 | 18 | 1,6 | 2,9 | cytoplasm | cytoplasm | eukaryote (human) | eukaryote (human) |
| **1gcq** | 56 | 69 | 26 | 8 | 5,5 | 4,7 | cytoplasm | cytoplasm | eukaryote (human) | eukaryote (mouse) |
| **1ghq** | 294 | 130 | complete | 13 | 0,5 | 1,9 | secreted, extracellular | extracellular | eukaryote (human) | eukaryote (human) |
| **1gl4** | 273 | 89 | 22 | 2 | 2,0 | 7,2 | secreted, extracellular | secreted, extracellular | eukaryote (mouse) | eukaryote (mouse) |
| **1glb** | 150 | 497 | 89 | complete | 1,7 | 0,0 | cytoplasm | cytoplasm | bacteria (escherichia) | bacteria (escherichia) |
| **1go4** | 195 | 87 | 96 | 12 | 6,4 | 10,9 | nucleus | nucleus | eukaryote (human) | eukaryote (human) |
| **1gpq** | 128 | 129 | complete | complete | 3,9 | 4,6 | periplasm | secreted, extracellular | bacteria (escherichia) | eukaryote (chicken) |
| **1gpw** | 253 | 200 | complete | complete | 2,1 | 2,8 | cytoplasm | cytoplasm | bacteria (thermotoga) | bacteria (thermotoga) |
| **1gzs** | 178 | 165 | 95 | 69 | 4,8 | 5,7 | cytoplasm | cytoplasm | eukaryote (human) | bacteria (salmonella) |
| **1h2s** | 225 | 60 | 94 | 11 | 1,7 | 6,4 | plasma membrane | plasma membrane | archea (natronomonas) | archea (natronomonas) |
| **1hx1** | 377 | 112 | 58 | 32 | 1,6 | 5,4 | cytoplasm | cytoplasm | eukaryote (bovine) | eukaryote (human) |
| **1i1r** | 301 | 167 | 34 | 82 | 0,7 | 2,4 | extracellular | secreted, extracellular | eukaryote (human) | virus (herpesvirus) |
| **1i8l** | 199 | 118 | 78 | 63 | 1,9 | 4,1 | extracellular | extracellular | eukaryote (human) | eukaryote (human) |
| **1im3** | 275 | 95 | 81 | 48 | 1,6 | 4,3 | extracellular | ER | eukaryote (human) | virus (cytomegalovirus) |
| **1ira** | 311 | 145 | 56 | 95 | 2,6 | 5,5 | extracellular | secreted, extracellular | eukaryote (human) | eukaryote (human) |
| **1itb** | 310 | 153 | 56 | complete | 3,5 | 7,6 | extracellular | secreted, extracellular | eukaryote (human) | eukaryote (human) |
| **1ixs** | 315 | 50 | complete | 26 | 1,2 | 7,9 | cytoplasm | cytoplasm | bacteria (thermus) | bacteria (thermus) |
| **1j2j** | 165 | 41 | 92 | 6 | 1,9 | 8,0 | Golgi | Golgi | eukaryote (mouse) | eukaryote (human) |
| **1jch** | 468 | 84 | 85 | complete | 2,7 | 13,7 | cytoplasm | cytoplasm | bacteria (escherichia) | bacteria (escherichia) |
| **1jiw** | 470 | 105 | complete | complete | 1,8 | 6,6 | secreted, extracellular | periplasm | bacteria (pseudomonas) | bacteria (pseudomonas) |
| **1jk9** | 243 | 153 | complete | complete | 2,9 | 5,4 | cytoplasm | cytoplasm | eukaryote (yeast) | eukaryote (yeast) |
| **1jtd** | 262 | 273 | complete | complete | 2,4 | 2,4 | periplasm | secreted, extracellular | bacteria (escherichia) | bacteria (streptomyces) |
| **1k5d** | 206 | 146 | 96 | 73 | 6,4 | 9,0 | nucleus | nucleus | eukaryote (human) | eukaryote (human) |
| **1kac** | 185 | 124 | 32 | 36 | 1,7 | 2,8 | extracellular | extracellular | virus (adenovirus) | eukaryote (human) |
| **1kg0** | 188 | 136 | 79 | 61 | 2,4 | 3,0 | extracellular | extracellular | eukaryote (human) | virus (Epstein-Barr) |
| **1kgy** | 181 | 138 | 19 | 45 | 5,8 | 6,9 | extracellular | extracellular | eukaryote (mouse) | eukaryote (mouse) |
| **1klf** | 279 | 205 | complete | complete | 3,8 | 4,8 | fimbrium | periplasm | bacteria (escherichia) | bacteria (escherichia) |
| **1kps** | 159 | 156 | complete | 26 | 2,5 | 2,1 | cytoplasm | cytoplasm | eukaryote (human) | eukaryote (mouse) |
| **1lfd** | 167 | 87 | 90 | 10 | 2,4 | 4,6 | cytoplasm | cytoplasm | eukaryote (human) | eukaryote (rat) |
| **1lpb** | 448 | 85 | complete | 94 | 1,3 | 6,5 | secreted, extracellular | secreted, extracellular | eukaryote (human) | eukaryote (pig) |
| **1m27** | 104 | 61 | 81 | 11 | 2,5 | 5,5 | cytoplasm | cytoplasm | eukaryote (human) | eukaryote (human) |
| **1m9f** | 165 | 135 | complete | 58 | 1,8 | 1,7 | cytoplasm | cytoplasm | eukaryote (human) | virus (HIV-1) |
| **1mbx** | 142 | 87 | 19 | 82 | 3,9 | 4,5 | cytoplasm | cytoplasm | bacteria (escherichia) | bacteria (escherichia) |
| **1mq8** | 184 | 177 | 36 | 15 | 2,1 | 2,5 | extracellular | extracellular | eukaryote (human) | eukaryote (human) |
| **1mzw** | 173 | 31 | complete | 6 | 2,0 | 10,3 | nucleus | nucleus | eukaryote (human) | eukaryote (human) |
| **1nkp** | 88 | 83 | 20 | 52 | 9,1 | 10,1 | nucleus | nucleus | eukaryote (human) | eukaryote (human) |
| **1npe** | 263 | 164 | 22 | 10 | 2,3 | 2,9 | secreted, extracellular | secreted, extracellular | eukaryote (mouse) | eukaryote (mouse) |
| **1nql** | 612 | 48 | 52 | 91 | 0,6 | 7,2 | extracellular | extracellular | eukaryote (human) | eukaryote (human) |
| **1ohz** | 140 | 56 | 8 | 5 | 3,6 | 8,7 | cell wall | cell wall | bacteria (clostridium) | bacteria (clostridium) |
| **1oo0** | 144 | 92 | complete | 56 | 5,8 | 9,4 | nucleus | nucleus | eukaryote (fruit fly) | eukaryote (fruit fly) |
| **1oqd** | 144 | 39 | 95 | 21 | 2,4 | 8,6 | extracellular | extracellular | eukaryote (human) | eukaryote (human) |
| **1oqe** | 144 | 31 | 95 | 17 | 2,6 | 9,9 | extracellular | extracellular | eukaryote (human) | eukaryote (human) |
| **1ory** | 119 | 40 | 96 | 8 | 7,6 | 21,5 | flagellum | flagellum | bacteria (aquifex) | bacteria (aquifex) |
| **1oxb** | 166 | 124 | complete | 10 | 3,6 | 4,7 | cytoplasm | cytoplasm | eukaryote (yeast) | eukaryote (yeast) |
| **1pqz** | 238 | 99 | 62 | complete | 3,4 | 7,9 | extracellular | secreted, extracellular | virus (cytomegalovirus) | eukaryote (mouse) |
| **1puf** | 77 | 73 | 28 | 17 | 2,3 | 3,0 | nucleus | nucleus | eukaryote (mouse) | eukaryote (human) |
| **1pvh** | 201 | 169 | 22 | 94 | 2,1 | 2,5 | extracellular | secreted, extracellular | eukaryote (human) | eukaryote (human) |
| **1pxv** | 183 | 111 | complete | complete | 3,8 | 6,3 | cytoplasm | cytoplasm | bacteria (staphylococcus) | bacteria (staphylococcus) |
| **1qa9** | 102 | 95 | 31 | 46 | 3,8 | 5,0 | extracellular | extracellular | eukaryote (human) | eukaryote (human) |
| **1qav** | 115 | 90 | 8 | 18 | 4,8 | 6,0 | cytoplasm | cytoplasm | eukaryote (rat) | eukaryote (mouse) |
| **1r4a** | 165 | 51 | 92 | 2 | 1,5 | 4,6 | cytoplasm | cytoplasm | eukaryote (rat) | eukaryote (human) |
| **1s3s** | 441 | 118 | 55 | 32 | 1,1 | 4,0 | cytoplasm | cytoplasm | eukaryote (mouse) | eukaryote (rat) |
| **1sq0** | 202 | 266 | 10 | 44 | 3,4 | 1,3 | secreted, extracellular | extracellular | eukaryote (human) | eukaryote (human) |
| **1sv0** | 77 | 82 | 11 | 46 | 4,6 | 4,6 | nucleus | nucleus | eukaryote (fruit fly) | eukaryote (fruit fly) |
| **1syx** | 135 | 62 | 95 | 18 | 2,5 | 4,2 | nucleus | cytoplasm | eukaryote (human) | eukaryote (human) |
| **1t0f** | 264 | 49 | complete | 9 | 3,0 | 16,9 | cytoplasm | cytoplasm | bacteria (escherichia) | bacteria (escherichia) |
| **1t9g** | 387 | 228 | complete | 90 | 1,2 | 1,8 | mitochondrion | mitochondrion | eukaryote (human) | eukaryote (human) |
| **1ta3** | 301 | 274 | complete | complete | 1,9 | 1,8 | secreted, extracellular | secreted, extracellular | eukaryote (aspergillus) | eukaryote (wheat) |
| **1taf** | 68 | 70 | 24 | 12 | 13,2 | 12,6 | nucleus | nucleus | eukaryote (fruit fly) | eukaryote (fruit fly) |
| **1tdq** | 271 | 126 | 20 | 6 | 2,0 | 3,8 | secreted, extracellular | secreted, extracellular | eukaryote (rat) | eukaryote (rat) |
| **1te1** | 274 | 190 | complete | complete | 1,9 | 3,0 | secreted, extracellular | secreted, extracellular | eukaryote (wheat) | eukaryote (penicillium) |
| **1tmq** | 470 | 117 | complete | 96 | 1,5 | 5,7 | cytoplasm | secreted, extracellular | eukaryote (yellow mealworm) | eukaryote (eleusine) |
| **1tnr** | 144 | 139 | 84 | 55 | 2,2 | 2,2 | secreted, extracellular | extracellular | eukaryote (human) | eukaryote (human) |
| **1txq** | 74 | 64 | 6 | 24 | 3,3 | 7,3 | cytoplasm | cytoplasm | eukaryote (human) | eukaryote (human) |
| **1tyg** | 242 | 65 | 95 | complete | 3,3 | 12,0 | cytoplasm | cytoplasm | bacteria (bacillus) | bacteria (bacillus) |
| **1u0s** | 118 | 86 | complete | 13 | 4,6 | 5,2 | cytoplasm | cytoplasm | bacteria (thermotoga) | bacteria (thermotoga) |
| **1uad** | 173 | 92 | 85 | 10 | 2,0 | 4,9 | cytoplasm | cytoplasm | eukaryote (human) | eukaryote (rat) |
| **1uw4** | 248 | 91 | 19 | 19 | 2,2 | 6,2 | cytoplasm | cytoplasm | eukaryote (human) | eukaryote (human) |
| **1v74** | 107 | 87 | 15 | complete | 5,5 | 5,4 | cytoplasm | cytoplasm | bacteria (escherichia) | bacteria (escherichia) |
| **1vet** | 122 | 118 | complete | 94 | 7,7 | 7,4 | cytoplasm | cytoplasm | eukaryote (mouse) | eukaryote (mouse) |
| **1w1i** | 728 | 349 | complete | 96 | 0,5 | 1,4 | extracellular | extracellular | eukaryote (human) | eukaryote (bovine) |
| **1wmh** | 83 | 82 | 14 | 24 | 4,9 | 5,3 | cytoplasm | cytoplasm | eukaryote (human) | eukaryote (human) |
| **1wmi** | 88 | 61 | complete | 91 | 13,2 | 18,2 | cytoplasm | cytoplasm | archea (pyrococcus) | archea (pyrococcus) |
| **1wq1** | 166 | 324 | 90 | 31 | 3,1 | 0,9 | cytoplasm | cytoplasm | eukaryote (human) | eukaryote (human) |
| **1wrd** | 98 | 76 | 20 | complete | 2,3 | 3,0 | cytoplasm | cytoplasm | eukaryote (human) | eukaryote (bovine) |
| **1wyw** | 216 | 79 | 53 | 81 | 3,0 | 8,4 | nucleus | nucleus | eukaryote (human) | eukaryote (human) |
| **1xg2** | 317 | 151 | complete | complete | 2,2 | 3,8 | cell wall | cell wall | eukaryote (tomato) | eukaryote (kiwi) |
| **1xk4** | 88 | 92 | 95 | 80 | 9,6 | 7,4 | cytoplasm | cytoplasm | eukaryote (human) | eukaryote (human) |
| **1xqs** | 264 | 165 | 73 | 26 | 3,3 | 4,9 | cytoplasm | cytoplasm | eukaryote (human) | eukaryote (human) |
| **1xtg** | 424 | 59 | 95 | 29 | 4,1 | 29,4 | secreted, extracellular | cytoplasm | bacteria (clostridium) | eukaryote (human) |
| **1xu1** | 137 | 38 | 94 | 13 | 3,2 | 7,3 | secreted, extracellular | extracellular | eukaryote (mouse) | eukaryote (human) |
| **1y4h** | 173 | 109 | complete | complete | 4,7 | 6,8 | cytoplasm | cytoplasm | bacteria (staphylococcus) | bacteria (staphylococcus) |
| **1ycs** | 191 | 193 | 49 | 17 | 2,6 | 2,8 | cytoplasm | cytoplasm | eukaryote (human) | eukaryote (human) |
| **1z0j** | 169 | 51 | 87 | 7 | 2,2 | 6,1 | cytoplasm | cytoplasm | eukaryote (mouse) | eukaryote (human) |
| **1z3e** | 119 | 67 | 91 | 21 | 4,5 | 7,9 | cytoplasm | cytoplasm | bacteria (bacillus) | bacteria (bacillus) |
| **1z5y** | 136 | 118 | 74 | 22 | 3,2 | 4,0 | periplasm | periplasm | bacteria (escherichia) | bacteria (escherichia) |
| **1zc3** | 173 | 109 | 85 | 15 | 2,9 | 3,6 | cytoplasm | cytoplasm | eukaryote (human) | eukaryote (rat) |
| **2a5d** | 187 | 163 | 96 | 94 | 3,3 | 2,9 | secreted, extracellular | Golgi | bacteria (vibrio) | eukaryote (human) |
| **2a78** | 207 | 170 | complete | 84 | 2,7 | 3,6 | cytoplasm | cytoplasm | eukaryote (human) | virus (bacteriophage) |
| **2b59** | 166 | 156 | 22 | 9 | 2,9 | 3,5 | secreted, extracellular | secreted, extracellular | bacteria (clostridium) | bacteria (clostridium) |
| **2bh1** | 238 | 68 | 58 | 14 | 1,8 | 5,3 | cytoplasm | cytoplasm | bacteria (vibrio) | bacteria (vibrio) |
| **2btf** | 374 | 139 | complete | complete | 1,6 | 5,1 | cytoplasm | cytoplasm | eukaryote (bovine) | eukaryote (bovine) |
| **2c1m** | 424 | 46 | 80 | 10 | 4,1 | 29,6 | nucleus | nucleus | eukaryote (mouse) | eukaryote (mouse) |
| **2ckh** | 225 | 72 | 35 | 77 | 3,8 | 9,0 | nucleus | nucleus | eukaryote (human) | eukaryote (human) |
| **2ey4** | 329 | 52 | complete | 87 | 2,6 | 16,8 | cytoplasm | cytoplasm | archea (pyrococcus) | archea (pyrococcus) |
| **2gy7** | 216 | 423 | 45 | 38 | 1,3 | 0,5 | secreted, extracellular | cytoplasm | eukaryote (human) | eukaryote (human) |
| **2sni** | 275 | 65 | complete | 78 | 2,8 | 7,8 | secreted, extracellular | secreted, extracellular | bacteria (bacillus) | eukaryote (barley) |
| **3fap** | 107 | 92 | complete | 4 | 1,7 | 2,0 | cytoplasm | cytoplasm | eukaryote (human) | eukaryote (human) |
| **3sic** | 275 | 108 | complete | 96 | 2,7 | 6,4 | secreted, extracellular | secreted, extracellular | bacteria (bacillus) | bacteria (streptomyces) |
| **3ygs** | 95 | 97 | 8 | 23 | 2,5 | 3,2 | cytoplasm | cytoplasm | eukaryote (human) | eukaryote (human) |
| **4cpa** | 307 | 37 | complete | 95 | 1,0 | 12,6 | secreted, extracellular | cytoplasm | eukaryote (bovine) | eukaryote (potato) |
|  |  |  |  |  |  |  |  |  |  |  |
| **59 pairs** | eukaryotic intracellular (cytosolic or nuclear) proteins | | | | |  |  |  |  |  |
| **22 pairs** | archeal and bacterial intracellular proteins | | | |  |  |  |  |  |  |
| **53 pairs** | organellar proteins, intra- and extracellular segments of transmembrane proteins and secreted proteins | | | | | | | |  |  |
