# Supplementary material for: Hydrophilicity Matching – A Potential Prerequisite for the Formation of Protein-Protein Complexes in the Cell
Source: PLoS One. 2010 Jun 17;5(6):e11169. doi: 10.1371/journal.pone.0011169 (PMC2887369; doi:10.1371/journal.pone.0011169)
Supplement: Table S2 — Average Pearson correlation coefficient estimates for various properties between binding partners in different subsets. (0.04 MB DOC) [file pone.0011169.s003.doc]

**Table S2.** Average Pearson correlation coefficient estimates <R> for various properties between binding partners in different subsets.

| **compared property** | **59 pairs^*^** | **81 pair^†^** | **53 pairs^‡^** | **134 pairs^§^** |
| --- | --- | --- | --- | --- |
| N | -0,068 | -0,180 | -0,158 | -0,119 |
| R_gyr_ | 0,245 | 0,058 | -0,175 | -0,032 |
| SASA | -0,023 | -0,179 | -0,154 | -0,150 |
| vol | -0,068 | -0,174 | -0,152 | -0,124 |
| HFE | -0,016 | -0,088 | -0,106 | -0,097 |
| EE | -0,014 | -0,122 | -0,112 | -0,069 |
| HFE/N | **0,564** | 0,494 | 0,102 | **0,537** |
| HFE/R_gyr_ | 0,045 | 0,017 | -0,010 | 0,034 |
| HFE/SASA | 0,463 | 0,413 | 0,098 | 0,429 |
| HFE/vol | **0,557** | 0,463 | 0,000 | 0,495 |
| EE/N | **0,616** | **0,548** | 0,116 | 0,453 |
| EE/R_gyr_ | 0,074 | 0,006 | -0,039 | 0,027 |
| EE/SASA | 0,296 | 0,320 | -0,040 | 0,251 |
| EE/vol | **0,630** | **0,565** | 0,180 | **0,506** |

^*^eukaryotic intracellular (nuclear and cytosolic) complexes, ^†^archeal, bacterial and eukaryotic intracellular (nuclear and cytosolic) complexes (includes the entire subset of 59 binary complexes), ^‡^archeal, bacterial and eukaryotic extracellular complexes, or intracellular complexes of organellar proteins or segments of transmembrane proteins, ^§^maximal set comprised of ^†^ and ^‡^; the values of R > 0.5 are bolded
