# Supplementary material for: Hydrophilicity Matching – A Potential Prerequisite for the Formation of Protein-Protein Complexes in the Cell
Source: PLoS One. 2010 Jun 17;5(6):e11169. doi: 10.1371/journal.pone.0011169 (PMC2887369; doi:10.1371/journal.pone.0011169)
Supplement: Table S3 — Comparison of the absolute values (nm2) of different types of solvent-accessible surface area (SASA) between the known binding partners within different subsets. (0.03 MB DOC) [file pone.0011169.s004.doc]

**Table S3. Comparison of the absolute values (nm^2^) of different types of solvent-accessible surface area (SASA) between the known binding partners within different subsets.**

|  | **59 pairs^*^** |  | **81 pair^†^** |  | **53 pairs^‡^** |  | **134 pairs^§^** |  |
| --- | --- | --- | --- | --- | --- | --- | --- | --- |
| **SASA type** | **ICC** | **p-value** | **ICC** | **p-value** | **ICC** | **p-value** | **ICC** | **p-value** |
| negatively charged | 0,50 | 0,470275 | 0,44 | 0,834244 | 0,52 | 0,338605 | 0,47 | 0,713511 |
| positively charged | 0,50 | 0,461502 | 0,45 | 0,815994 | 0,49 | 0,530152 | 0,46 | 0,786833 |
| total charged | 0,51 | 0,413542 | 0,45 | 0,797718 | 0,51 | 0,404612 | 0,47 | 0,704149 |
| hydrophilic | 0,49 | 0,522343 | 0,42 | 0,926614 | 0,43 | 0,841014 | 0,43 | 0,938887 |
| hydrophobic | 0,47 | 0,630909 | 0,41 | 0,959405 | 0,41 | 0,899141 | 0,42 | 0,964151 |

^*^eukaryotic intracellular (nuclear and cytosolic) complexes, ^†^archeal, bacterial and eukaryotic intracellular (nuclear and cytosolic) complexes (includes the entire subset of 59 binary complexes), ^‡^archeal, bacterial and eukaryotic extracellular complexes, or intracellular complexes of organellar proteins or segments of transmembrane proteins, ^§^maximal set comprised of ^†^ and ^‡^
