# Supplementary material for: Hydrophilicity Matching – A Potential Prerequisite for the Formation of Protein-Protein Complexes in the Cell
Source: PLoS One. 2010 Jun 17;5(6):e11169. doi: 10.1371/journal.pone.0011169 (PMC2887369; doi:10.1371/journal.pone.0011169)
Supplement: Table S4 — Comparison of different types of solvent-accessible surface area (SASA) normalized by total SASA between the known binding partners within different subsets. (0.03 MB DOC) [file pone.0011169.s005.doc]

**Table S4. Comparison of different types of solvent-accessible surface area (SASA) normalized by total SASA between the known binding partners within different subsets.**

|  | **59 pairs^*^** |  | **81 pair^†^** |  | **53 pairs^‡^** |  | **134 pairs^§^** |  |
| --- | --- | --- | --- | --- | --- | --- | --- | --- |
| **SASA type** | **ICC** | **p-value** | **ICC** | **p-value** | **ICC** | **p-value** | **ICC** | **p-value** |
| negatively charged | 0,49 | 0,525300 | 0,46 | 0,724072 | 0,49 | 0,511719 | 0,53 | 0,220921 |
| positively charged | 0,64 | 0,012873 | 0,60 | 0,027991 | 0,54 | 0,245783 | **0,63** | **0,000934** |
| total charged | 0,67 | 0,002326 | **0,72** | **0,000006** | 0,58 | 0,121046 | **0,73** | **0,000001** |
| hydrophilic | 0,59 | 0,068765 | 0,58 | 0,079322 | 0,78 | 0,001706 | **0,70** | **0,000120** |
| hydrophobic | 0,59 | 0,068292 | 0,58 | 0,078805 | 0,78 | 0,001765 | **0,70** | **0,000118** |

^*^eukaryotic intracellular (nuclear and cytosolic) complexes, ^†^archeal, bacterial and eukaryotic intracellular (nuclear and cytosolic) complexes (includes the entire subset of 59 binary complexes), ^‡^archeal, bacterial and eukaryotic extracellular complexes, or intracellular complexes of organellar proteins or segments of transmembrane proteins, ^§^maximal set comprised of ^†^ and ^‡^; the p-values < 0.001 are bolded
