# Supplementary figures and images for: Hydrophilicity Matching – A Potential Prerequisite for the Formation of Protein-Protein Complexes in the Cell
Source: PLoS One. 2010 Jun 17;5(6):e11169. doi: 10.1371/journal.pone.0011169 (PMC2887369; doi:10.1371/journal.pone.0011169)

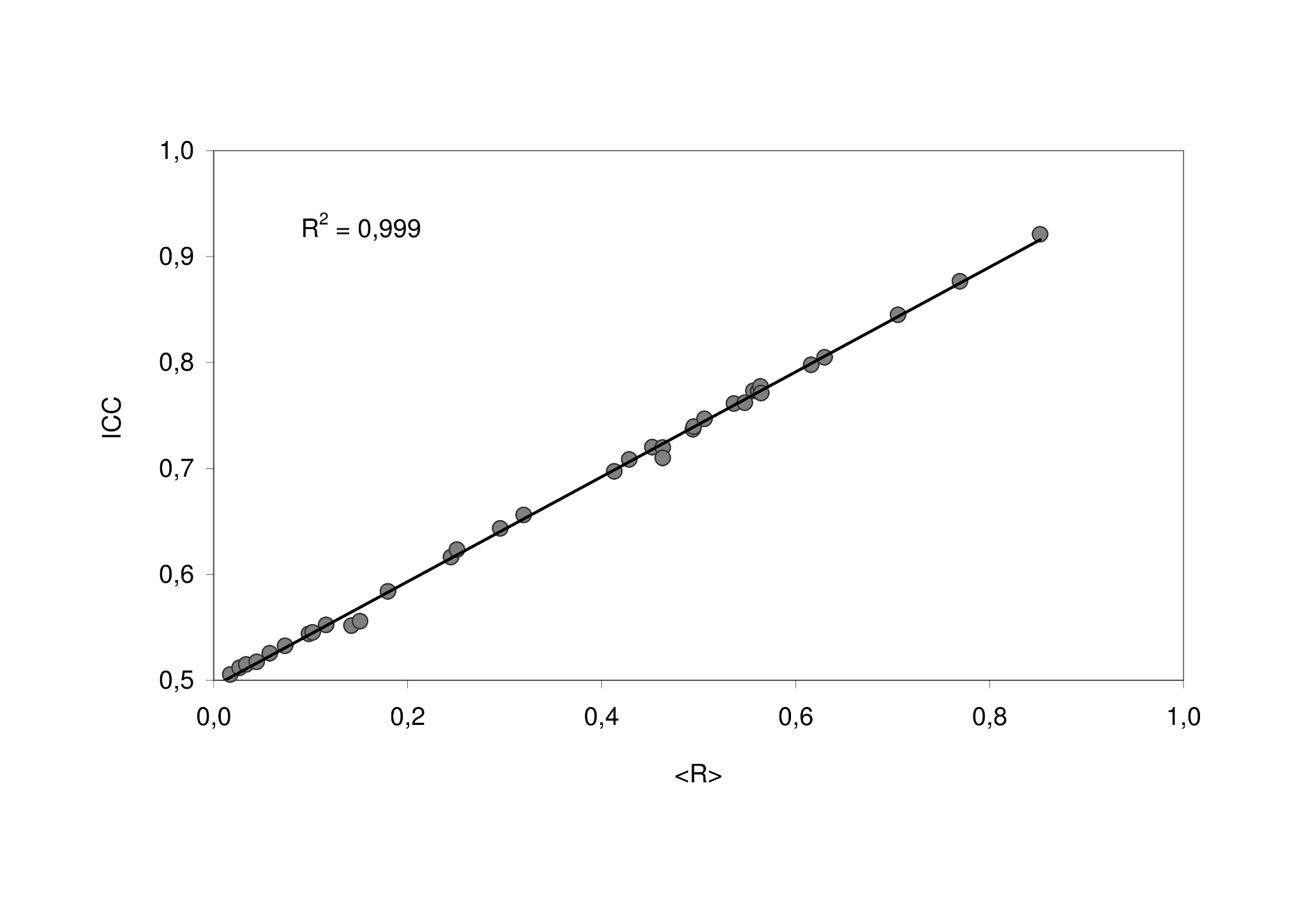

Supplement: Figure S1 — ICC vs Pearson R for various calculated properties. We show only the data points with ICC>0.5, indicating positive correlation in terms of R. The plotted R is the average obtained by 106 permutations of the members of each pair. (0.10 MB TIF) [file pone.0011169.s006.tif]

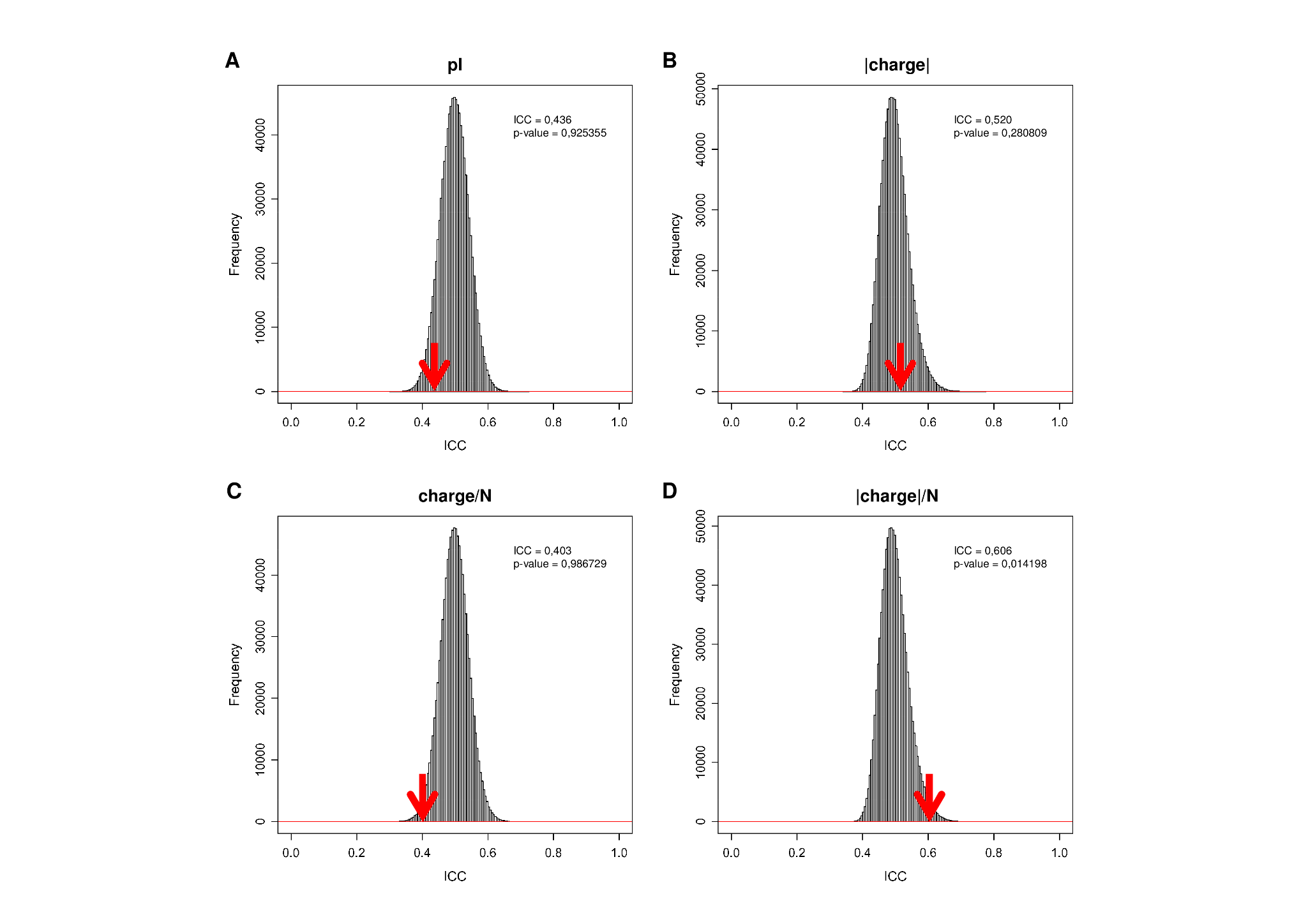

Supplement: Figure S2 — Comparison of ICCs calculated for naturally occuring binding partners and those obtained by a randomization procedure. The results are for the entire set of 268 eukaryotic proteins (134 pairs). The ICC values were calculated for (A) isoelectric point values (pI), (B) absolute values of charge (|charge|), (C) charge normalized by sequence length (charge/N), and (D) absolute values of charge normalized by sequence length of the partners (|charge|/N). The values of charge used were all at neutral pH. Red arrow denotes the value of the observed ICC for the known binding partners. (0.22 MB TIF) [file pone.0011169.s007.tif]

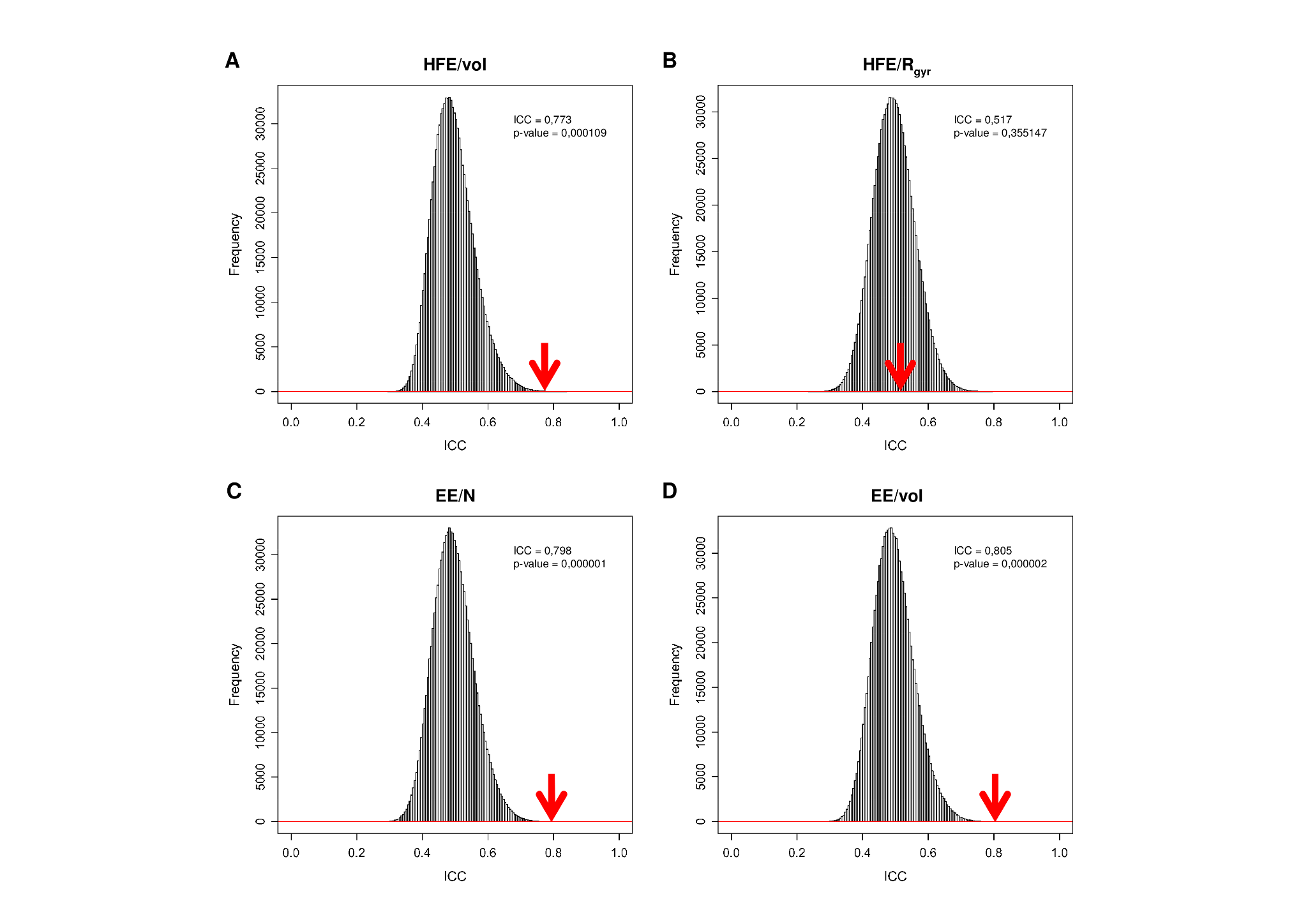

Supplement: Figure S3 — Comparison of ICCs calculated for naturally occuring binding partners and those obtained by a randomization procedure. The results are for a subset of 118 eukaryotic proteins (59 pairs) that interact in the cytoplasm or nucleoplasm. The ICC values were calculated for (A) the hydration free energy normalized by volume of the partners (HFE/vol), (B) HFE normalized by radius of gyration (HFE/Rgyr), (C) electrostatic energy normalized by sequence length (EE/N), and (D) electrostatic energy normalized by volume (EE/vol). Red arrow denotes the value of the observed ICC for the known binding partners. (0.29 MB TIF) [file pone.0011169.s008.tif]

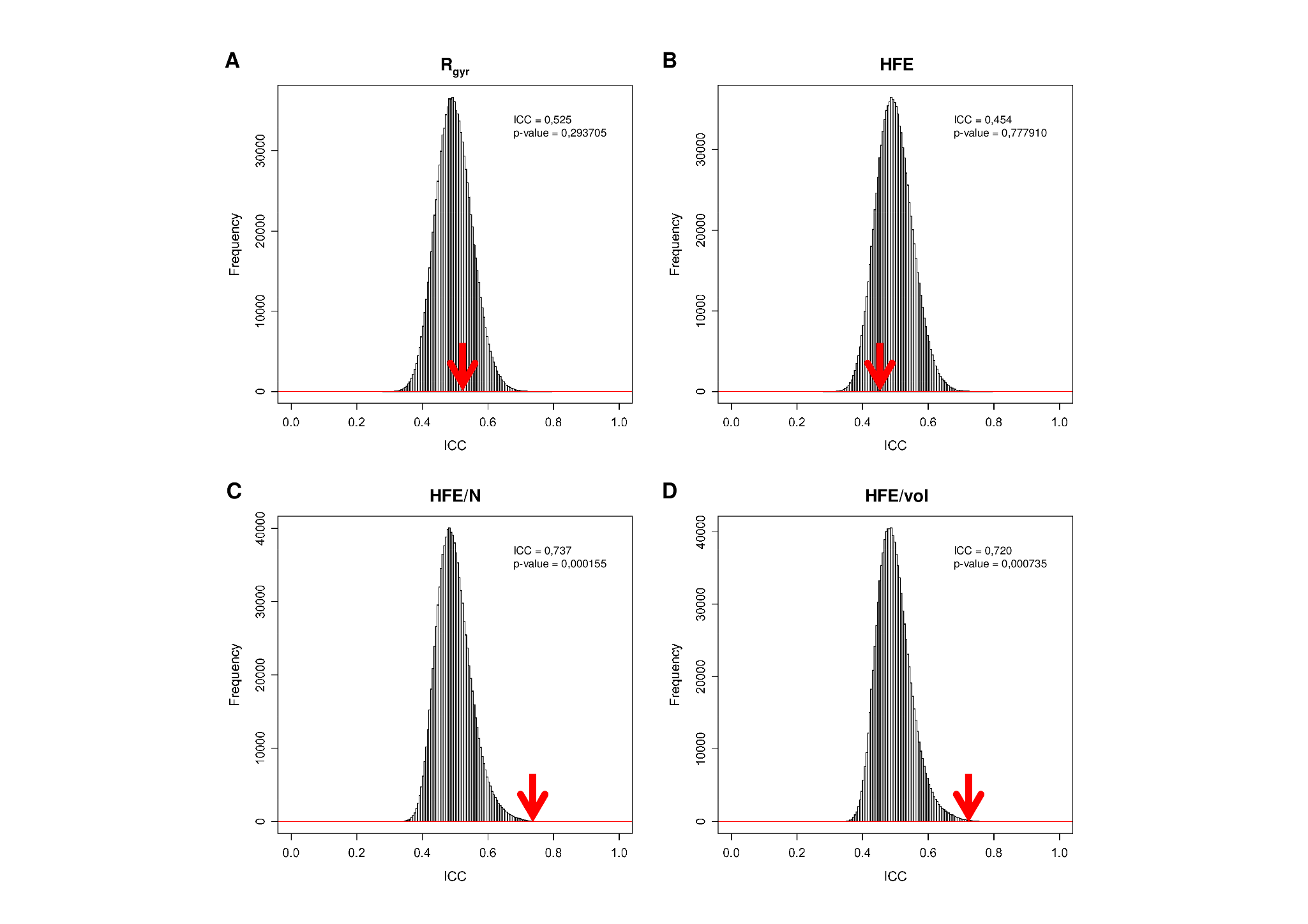

Supplement: Figure S4 — Comparison of ICCs calculated for naturally occuring binding partners and those obtained by a randomization procedure. The results are for a subset of 162 intracellular proteins from all three domains of life. The ICC values were calculated for (A) the radius of gyration (Rgyr), (B) hydration free energy (HFE), (C) HFE normalized by sequence length (HFE/N), and (D) HFE normalized by volume (HFE/vol). Red arrow denotes the value of the observed ICC for the known binding partners. (0.24 MB TIF) [file pone.0011169.s009.tif]

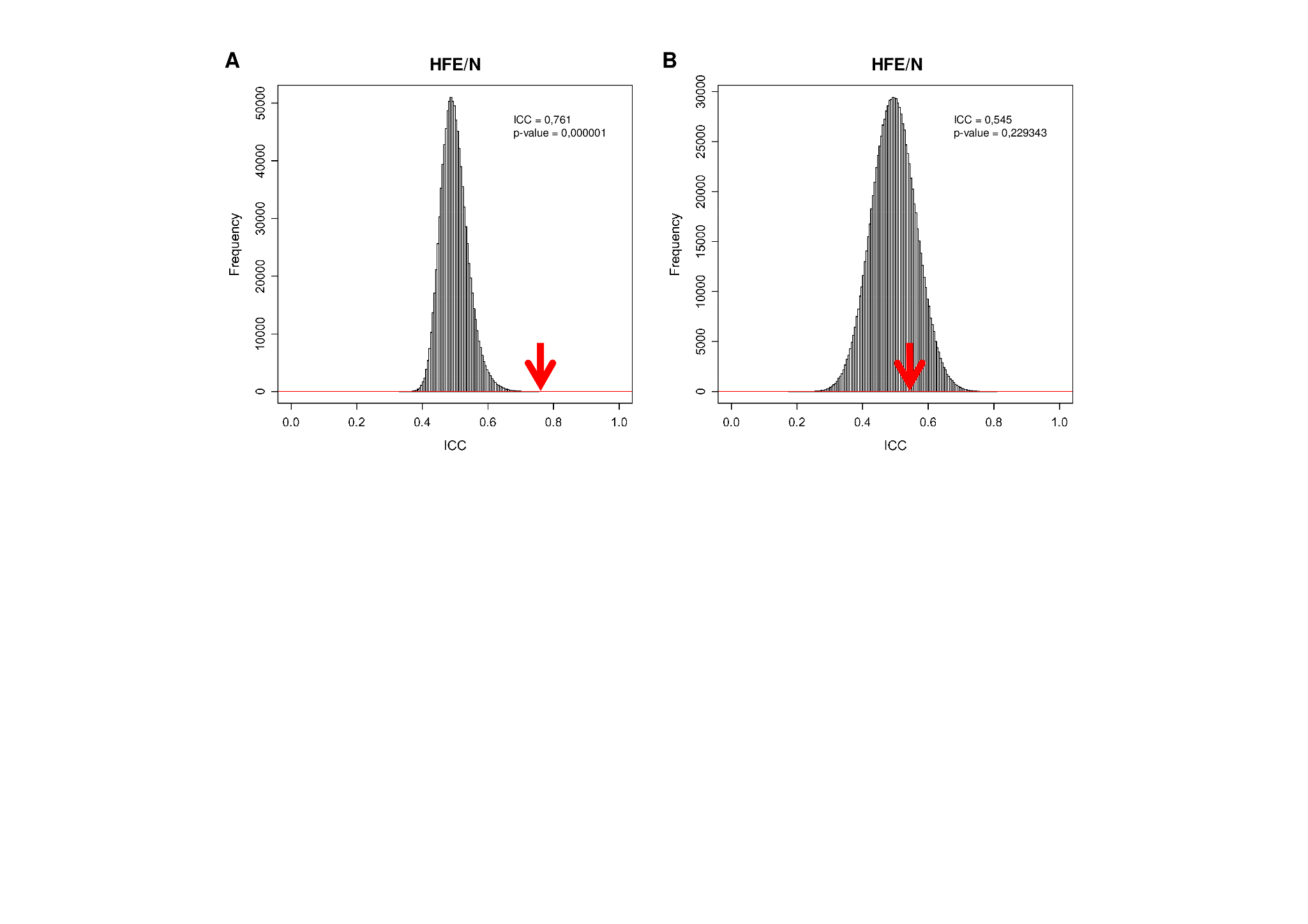

Supplement: Figure S5 — Comparison of ICCs calculated for naturally occuring binding partners and those obtained by a randomization procedure. The ICC values were calculated for the size-normalized hydration free energy (HFE/N) in (A) the maximal set with all analyzed proteins (268 proteins in total), and (B) set containing only organellar and secreted proteins, as well as intra- and extracellular segments of transmembrane proteins (106 proteins in total). Red arrow denotes the value of the observed ICC for the known binding partners. (0.14 MB TIF) [file pone.0011169.s010.tif]

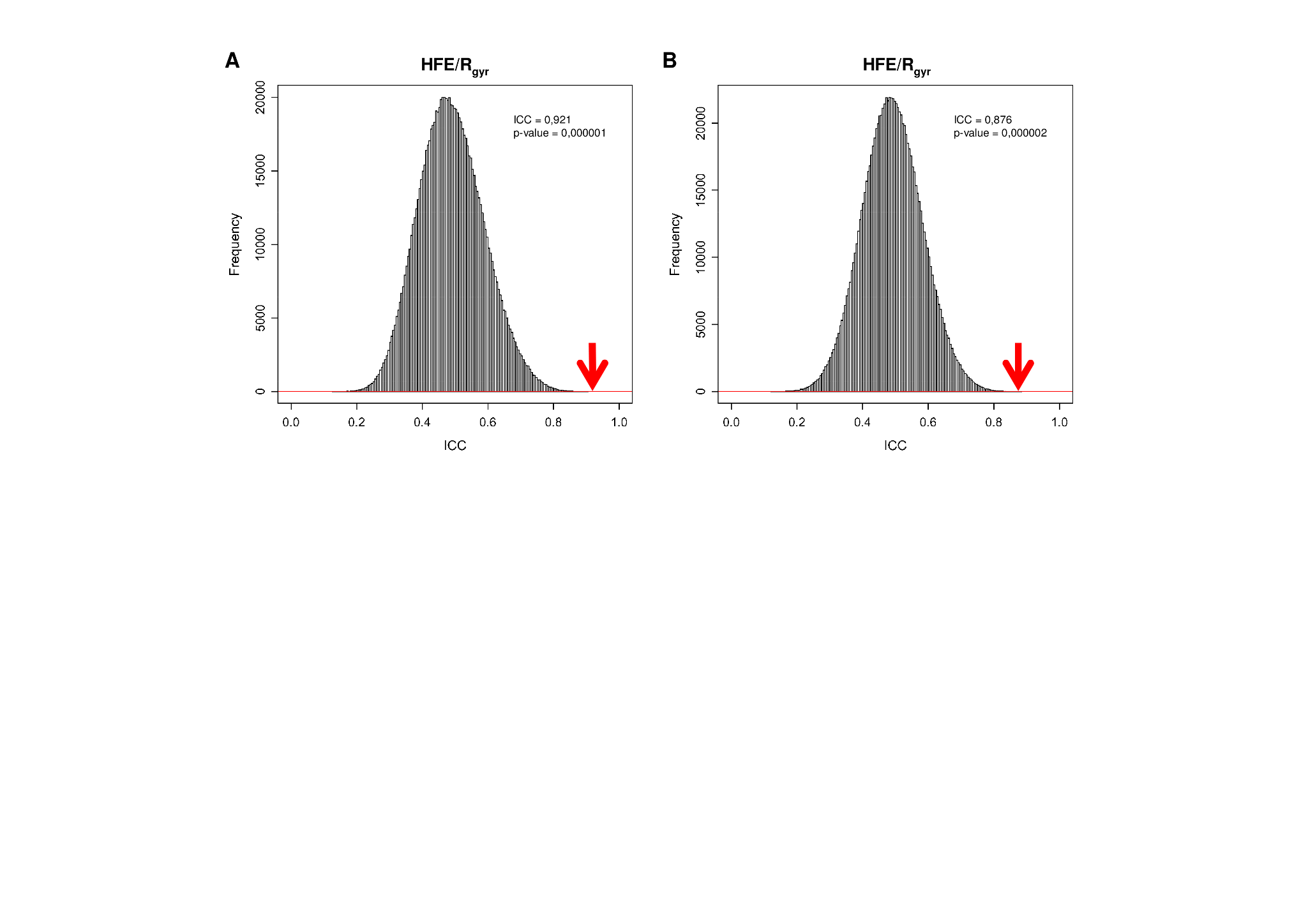

Supplement: Figure S6 — Comparison of ICCs calculated for naturally occuring binding partners and those obtained by a randomization procedure. The ICC values were calculated for the hydration free energy normalized by radius of gyration of the partners (HFE/Rgyr) for (A) 24 complexes, and (B) 28 complexes. Complexes were extracted by size-filtering of fragmented proteins with a criterion that both of the partners have less than 130 residues. Red arrow denotes the value of the observed ICC for the known binding partners. (0.21 MB TIF) [file pone.0011169.s011.tif]

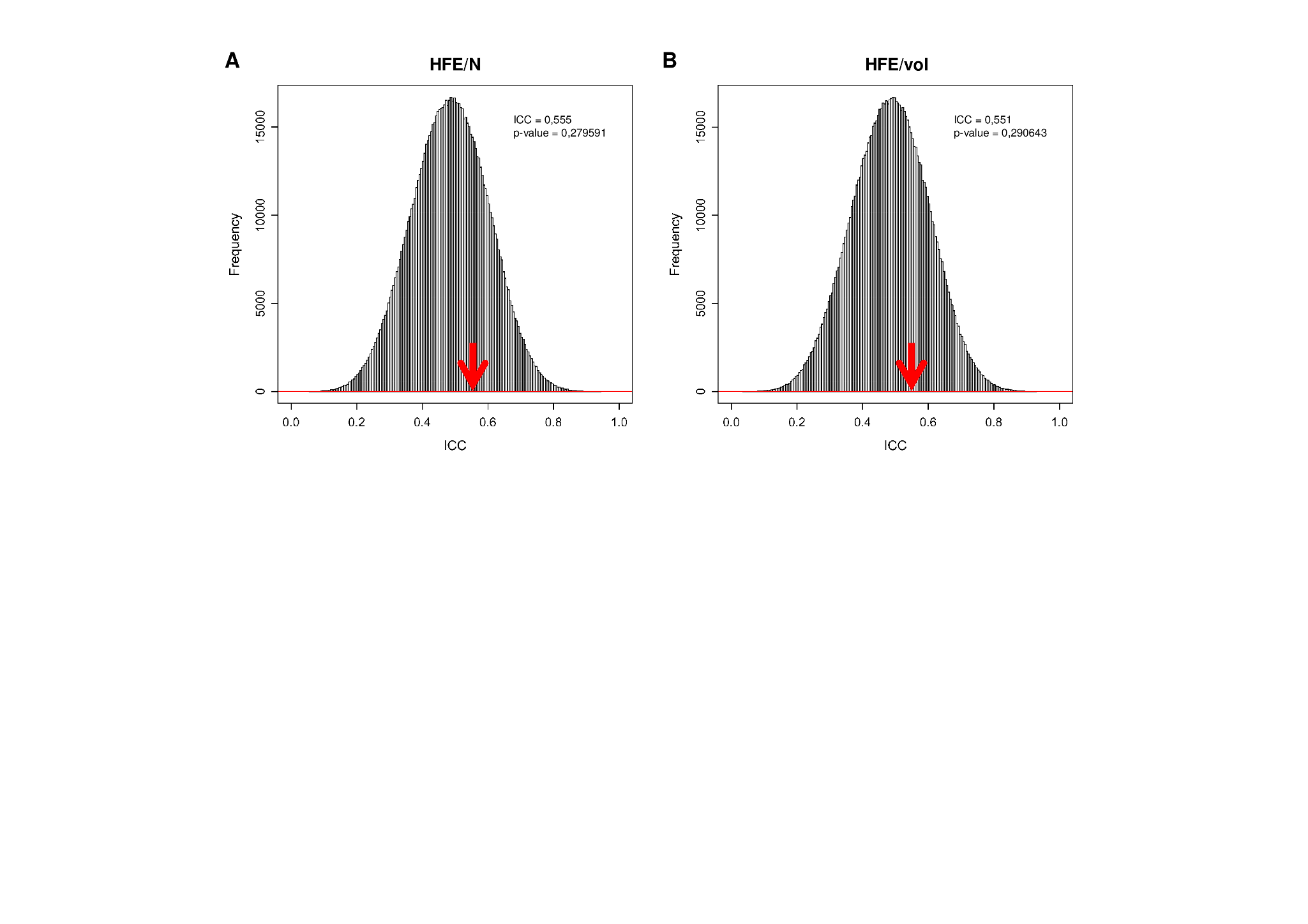

Supplement: Figure S7 — Comparison of ICCs calculated for naturally occuring binding partners and obtained by a randomization procedure. The results are for the set of complete proteins (17 complexes). The ICC values were calculated for (A) the hydration free energy normalized by sequence length (HFE/N), and (B) hydration free energy normalized by volume of the partners (HFE/vol). Red arrow denotes the value of the observed ICC for the known binding partners. (0.24 MB TIF) [file pone.0011169.s012.tif]

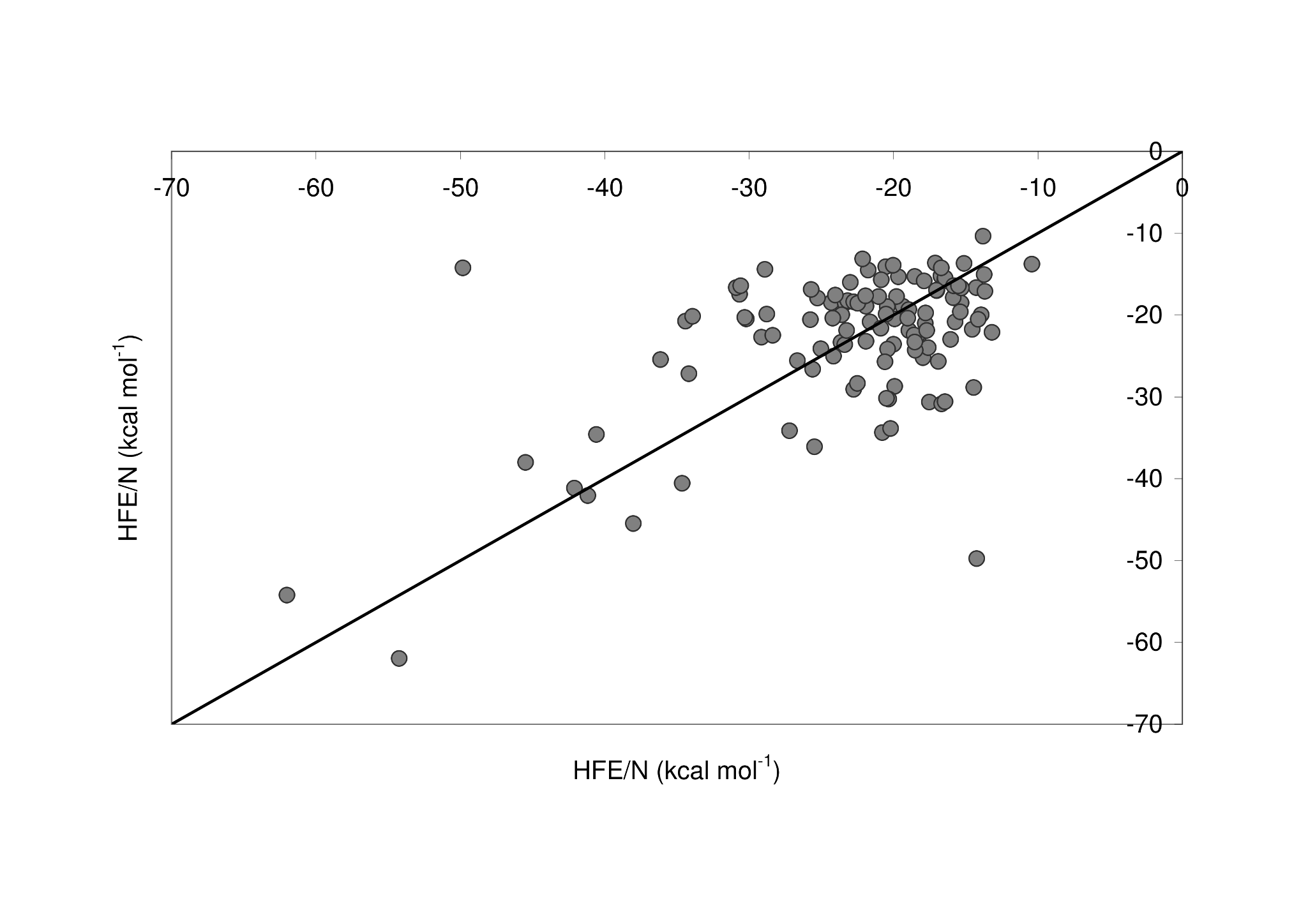

Supplement: Figure S8 — Symmetric scatter plot of the size-normalized hydration free energy (HFE/N). The data shown is for a subset of 118 eukaryotic proteins (59 pairs) that interact in the cytoplasm or nucleoplasm. Because it is impossible to uniquely assign each member of a given pair to either x or y axes, here we show both (x,y) and (y,x) possibilities for each point. (0.15 MB TIF) [file pone.0011169.s013.tif]

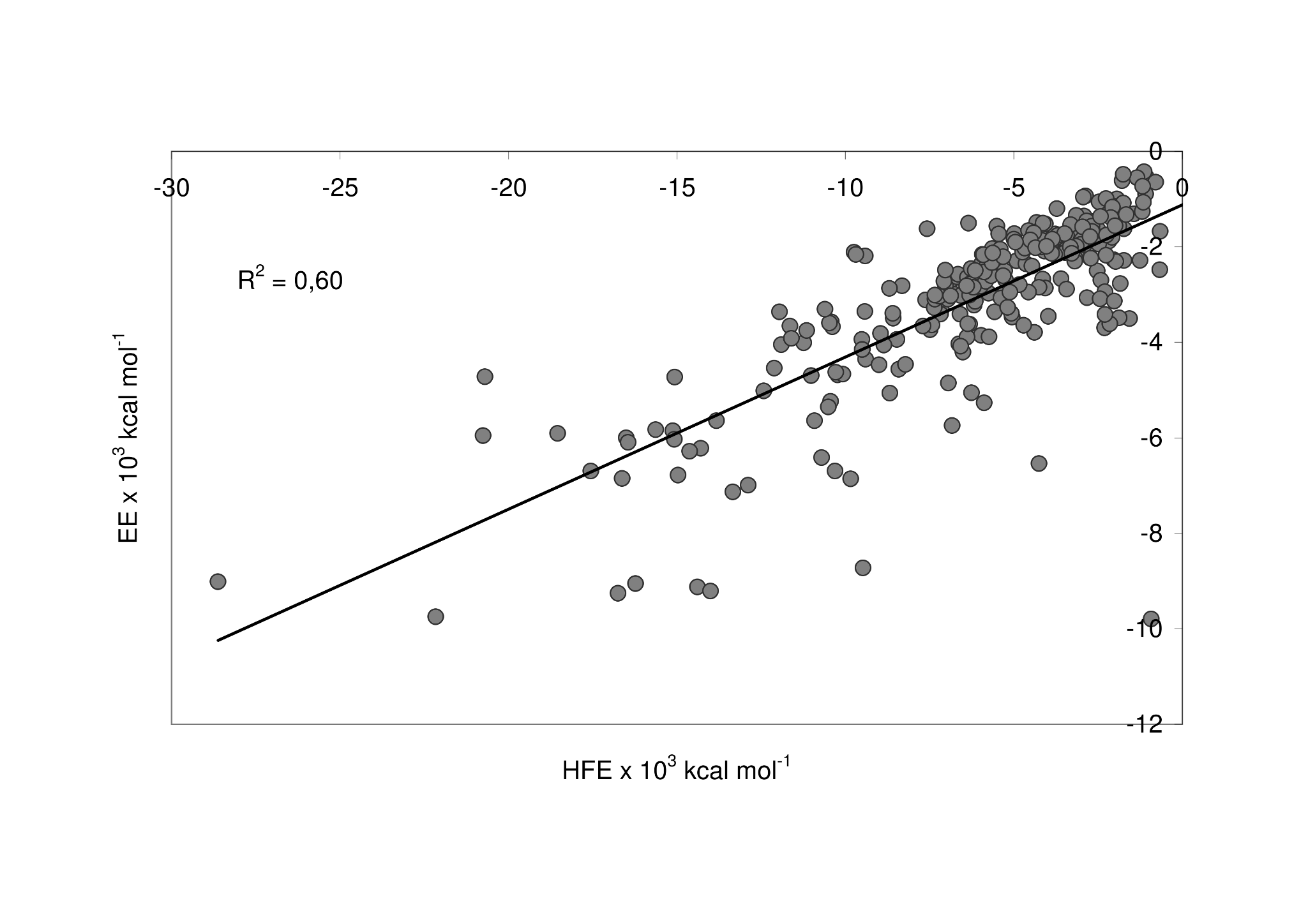

Supplement: Figure S9 — Electrostatic energy (EE) vs hydration free energy (HFE). The values shown are average HFE and EE calculated for all analyzed proteins (268 proteins in total). (0.18 MB TIF) [file pone.0011169.s014.tif]
